# Up-regulation of apoptotic- and cell survival-related gene pathways following exposures of western corn rootworm to *B. thuringiensis* crystalline pesticidal proteins in transgenic maize roots

**DOI:** 10.1186/s12864-021-07932-4

**Published:** 2021-09-04

**Authors:** Brad S. Coates, Emeline Deleury, Aaron J. Gassmann, Bruce E. Hibbard, Lance J. Meinke, Nicholas J. Miller, Jennifer Petzold-Maxwell, B. Wade French, Thomas W. Sappington, Blair D. Siegfried, Thomas Guillemaud

**Affiliations:** 1 USDA-ARS, Corn Insects & Crop Genetics Research Unit, 103 Genetics Laboratory, Iowa State University, Ames, IA 50011 USA; 2 University of Florida, Gainesville, FL USA; 3 INRAE, CNRS, Université Côte d’Azur, ISA, France; 4 Department of Entomology, Iowa State University, Ames, IA USA; 5 USDA-ARS, Plant Genetics Research Unit, Columbia, MO USA; 6 Department of Entomology, University of Nebraska, Lincoln, NE USA; 7 Illinois Institute of Technology, Chicago, IL USA; 8 USDA-ARS, North Central Agricultural Research Laboratory, Brookings, SD USA

**Keywords:** Diabrotica, Apoptosis, Cell fate, Stress response, Pesticidal proteins, Gene expression

## Abstract

**Background:**

Resistance of pest insect species to insecticides, including *B. thuringiensis* (Bt) pesticidal proteins expressed by transgenic plants, is a threat to global food security. Despite the western corn rootworm, *Diabrotica virgifera virgifera*, being a major pest of maize and having populations showing increasing levels of resistance to hybrids expressing Bt pesticidal proteins, the cell mechanisms leading to mortality are not fully understood.

**Results:**

Twenty unique RNA-seq libraries from the Bt susceptible *D. v. virgifera* inbred line Ped12, representing all growth stages and a range of different adult and larval exposures, were assembled into a reference transcriptome. Ten-day exposures of Ped12 larvae to transgenic Bt Cry3Bb1 and Gpp34/Tpp35Ab1 maize roots showed significant differential expression of 1055 and 1374 transcripts, respectively, compared to cohorts on non-Bt maize. Among these, 696 were differentially expressed in both Cry3Bb1 and Gpp34/Tpp35Ab1 maize exposures. Differentially-expressed transcripts encoded protein domains putatively involved in detoxification, metabolism, binding, and transport, were, in part, shared among transcripts that changed significantly following exposures to the entomopathogens *Heterorhabditis bacteriophora* and *Metarhizium anisopliae*. Differentially expressed transcripts in common between Bt and entomopathogen treatments encode proteins in general stress response pathways, including putative Bt binding receptors from the ATP binding cassette transporter superfamily. Putative caspases, pro- and anti-apoptotic factors, as well as endoplasmic reticulum (ER) stress-response factors were identified among transcripts uniquely up-regulated following exposure to either Bt protein.

**Conclusions:**

Our study suggests that the up-regulation of genes involved in ER stress management and apoptotic progression may be important in determining cell fate following exposure of susceptible *D. v. virgifera* larvae to Bt maize roots. This study provides novel insights into insect response to Bt intoxication, and a possible framework for future investigations of resistance mechanisms.

**Supplementary Information:**

The online version contains supplementary material available at 10.1186/s12864-021-07932-4.

## Background

The efficacy of pesticidal agents that control feeding damage on agriculturally-important crop plants become reduced following repeated exposures and selection for resistance within target arthropod pest populations [1–3]. A diversity of bacterial pore-forming pesticidal proteins have been described including *B. thuringiensis* (Bt) crystalline (Cry), toxin-10 like (Tpp) and AeGerolysin like pesticidal proteins (Gpp) [4]. Transgenic maize hybrids that express pesticidal proteins are widely used by growers in the United States and several other countries worldwide [5]. The number of insect species with documented resistance continues to increase [3, 6].

The western corn rootworm, *Diabrotica virgifera virgifera* (Insecta: Coleoptera: Chrysomelidae), causes extensive damage to cultivated maize, *Zea mays,* throughout the major production regions of North America and Europe [7, 8]. This univoltine species diapauses over the winter months as eggs in the soil. At high population densities, maize root feeding by larvae which hatch during early summer can reduce yields through both physiological and mechanical damage [9, 10]. Adult beetles emerge and feed mainly on maize silk and pollen. Larval damage was historically controlled by soil insecticides applied at planting [11]. In some regions, adults are sprayed aerially with insecticides to reduce egg-laying and hence larval populations the following year [12]. After its first detection in 2009 [13], a high proportion of *D. v. virgifera* larvae in field populations in the United States now show resistance to Cry3Bb1 [14] along with cross-resistance to the structurally similar mCry3A [15] and eCry3.1Ab proteins expressed by maize hybrids [16, 17]. Resistance to transgenic Cry34/35Ab1 (Gpp34/Tpp35Ab1 according to new nomenclature by Crickmore et al. (2021)) [4] maize is also documented in *D. v. virgifera* field populations, but these phenotypes show no cross-resistance to Cry3 proteins [18–20]. Analogous lack of Gpp34/Tpp35Ab1cross-resistance with mCry3Aa was shown in laboratory selected *D. v. virgifera* [21]. This resistance occurred to transgenic hybrids that express a “low-dose” of Bt proteins [22] and adversely impacts crop production [23, 24].

Ingested pesticidal proteins cause disruption of gut cell integrity in susceptible insects, leading to lethargic behavior, cessation of feeding, and death [25]. Midgut cells of susceptible *D. v. virgifera* larvae fed diet containing Cry3Aa1 and Gpp34/Tpp35Ab1 swell and shed microvilli and other cell debris into the gut lumen [26]. A proposed mode of action involves sequential binding of Bt pesticidal proteins to membrane-bound protein receptors on the apical side of midgut epithelial cell (enterocyte) membranes [27–29]. Within this model, receptor binding precedes the formation of an ion pore channel that generates an osmotic imbalance due to an influx of extracellular Ca^2+^ [30], leakage of gut contents, and eventual death of the insect. This model further proposes that ingested monomeric Cry toxins interact with the midgut apical membrane-bound receptor protein, cadherin, causing a conformational change in the pesticidal protein that leaves it liable to cleavage by gut proteases. Cleavage in turn facilitates subsequent oligomerization into a pre-pore structure [31] which inserts into the enterocyte membrane following interactions with cell surface aminopeptidase N [32] or alkaline phosphatase [33]. ATP binding cassette (ABC) transporters are also implicated as receptors for Cry proteins [34–36], and hypothesized to facilitate Cry protein oligomerization and membrane pore insertion [37, 38]. Additional gut proteins have been identified as putative Bt protein receptors in insects [39–42], including the glycosyl hydrolase, α–amylase, from *Tenebrio molitor* [43], but their roles in Bt intoxication remains unknown.

An alternative model for Cry protein mode of action proposes a Mg^2+^-dependent G protein-mediated cell signalling pathway. In this model, binding of Cry proteins to cadherin directly triggers an intracellular adenylate cyclase signalling cascade that activate protein kinase A (PKA) and cell death (apoptosis) [44, 45]. This model hypothesizes a mechanism independent of other receptors or requirments for pore formation [46].

Mechanisms of resistance often implicate structural or functional changes in a midgut receptor protein that putatively disrupts the Bt mode of action, but are mainly from studies on Bt resistant species of Lepidoptera. This includes alteration of cadherin receptor protein structure by transposon-mediated insertional knockout or point mutations, that result in reduced Cry1A binding [47–50]. An amino acid change in a transmembrane domain of tetraspanin 1 is associated with *Helicoverpa armigera* resistance to Cry1Ac [51]. Cry1A resistance is also associated with reduced expression of one or more aminopeptidase N paralog in *Spodoptera exigua* [52], *Trichoplusia ni* [53] and *Ostrinia nubilalis* [54], and alkaline phosphatase in *Heliothis virescens* [55]. The ABC transporter subfamily C member, *abcc2*, is linked or associated with lepidopteran resistance to Cry1Ac in *H. virescens* [34], *Plutella xylostella* [56], *Bombyx mori* [57], and *T. ni* [58], Cry2Ab2 in *Pectinophora possypiella* [59], and Cry1Fa in *O. nubilalis* [60] and *Spodoptera frugiperda* [61]. An ABC subfamily B member in in the coleopteran *Chrysomela tremula* is linked to Cry3Aa resistance, and is capable of mediating cell disruption via ectopic expression in *S. frugiperda Sf9* cells [62]. An *abcb* gene is also located in proximity to a single QTL for Cry3Bb1 resistance in *D. v. virgifera* [63].

Alternatively, enhanced repair of damaged midgut cells in response to Cry protein-mediated damage contributes to Cry1Ac resistance in *H. virescens* [64, 65], suggesting that stress-induced cell regeneration or degradation mechanisms are involved in physiological responses [66]. Conversely, transcripts encoding proteins involved in apoptosis (programmed cell death) are significantly up-regulated in *Manduca sexta* cells by Cry1Ac [67], and in the midgut of *S. exigua* by exposure to the Bt vegetative insecticidal protein (Vip) 3A protein [68]. Apoptosis was induced in *Choristoneura fumiferana* cells by a mechanism involving the mitogen activated protein kinase (MAPK) protein p38 following Cry1Ac exposure [69], and RNAi-mediated knockdown of MAPK p38 in *Chilo suppressalis* led to increased larval susceptibility to Cry1Ca [70].

Despite these insights, Bt mode(s) of action, mechanisms of cellular intoxication, and intracellular responses are not fully understood. In *D. v. virgifera*, mCry3A binding to midgut receptors is reduced among larvae selected for mCry3A resistance [21]. Kinetic data demonstrate that Cry3Bb binds strongly to specific domains of the cadherin protein and enhances toxicity in *D. v. virgifera* [71] and other Coleoptera [72]. However, cadherin is not considered a receptor in vivo since RNAi-based transcript knockdown does not alter *D. v. virgifera* susceptibility to Cry3Aa or Gpp34/Tpp35Ab1 [73]. Estimates of differential gene expression shows no significant induction of cadherin in susceptible compared to resistant larvae fed Cry3Bb1 [74], eCry3.1Ab [75], or between susceptible larvae exposed and not exposed to Cry3Bb1 [74] or Gpp34/Tpp35Ab1 [76]. Although a suite of ABC transporters and aminopeptidase N transcripts are differentially-regulated in constitutive or induced fashions between Bt resistant and susceptible *D. v. virgifera* larvae to Cry3Bb1 [74] and eCry3.1Ab [75], paralogs are also differentially-regulated in susceptible larvae exposed to Cry3Bb1 and Gpp34/Tpp35Ab1 [74, 76]. Furthermore, these transcriptome-wide comparisons have implicated a relatively large number of differentially expressed transcripts including those encoding proteins in general stress response pathways (e.g. cytochrome P450 monooxygenases, esterases, oxidases, and peroxidases) and those with transporter function [74].

A better understanding of how Bt intoxication affects gene expression among susceptible arthropods may reveal points at which mechanistic disruption could lead to resistance. To this end, we developed an inbred strain of Bt susceptible *D. v. virgifera*, Ped12, and used it for assembly of a comprehensive reference transcriptome, which was then applied to estimate transcript quantity differences following exposures to Cry3Bb1, Gpp34/Tpp35Ab1, and non-Bt maize within a common genetic background. Furthermore, transcripts differently expressed by Ped12 larvae following exposures to one or both Cry3Bb and Gpp34/Tpp35Ab1 maize, and exposures to an entomopathogenic fungus and nematode, were associated with generalized stress and immune response pathways. A filtered set of differentially expressed transcripts not shared with entomopathogens encoding candidate Bt receptor proteins, metabolic and detoxification enzymes, and proteins putatively determining cell fate (pro-survival or -death) were focused upon. This study contributes to an understanding of mechanisms potentially involved in determining cell fate (death or survival) which may inform future research into processes involved in Bt intoxication or resistance mechanisms.

## Results

### Samples, treatments, and collections

Samples of Ped12 *D. v. virgifera* were collected from all developmental stages and different exposure conditions to create a reference transcriptome assembly (C1 to C20; Table 1). Among replicate treatments used in the downstream analysis of differential gene expression (T1 to T8; Table 2), Ped12 2nd instars were recovered from transgenic Cry3Bb1 (VT3; T8) and Gpp34/Tpp35Ab1 (Hx; T5) maize treatments after 48 h exposure. Approximately 1/3 of larvae were dissected from inside Cry3Bb1-expressing roots (T8), whereas none were found burrowing inside the roots of Gpp34/Tpp35Ab1 hybrid maize (T5). All *D. v. virgifera* larvae in the control non-Bt maize Corteva Pioneer hybrid 38B85 treatment (T7) were feeding within roots. Larvae exposed for 48 h to *M. anisopliae* (T3) and *H. bacteriophora* (T6) were lethargic, but none were moribund.

**Table 1:** Developmental stages and conditions (single replicates) in the normalized reference transcriptome from *Diabrotica virgifera virgifera* inbred line Ped12

ID	Stage	Stage or condition	Description
C01	Adult	Heat (30 min at 37 °C)	Heat shock
C02	Adult	Starved 72 h	Starvation
C03	Adult	Mated females	Post-mating
C04	Adult	Mated males	Post-mating
C05	Adult	Unmated females	Virgin
C06	Adult	Unmated males	Virgin
C07	1st instar	Larval development; early-stage	Larval instar
C08	2nd instar	Larval development; mid-stage	Larval instar
C09	3rd instar	Larval development; late-stage	Larval instar
C10	Adult	Unmated adults; mixed sex	Adult (early; virgin)
C11	Egg (< 1 day)	Embryonic; early development	Embryonic (early)
C12	Egg (12–16 day)	Embryonic; late development	Embryonic (late)
C13	2nd and 3rd instar	Exposed to mCry3A maize Event MIR604 ^A^	Insecticidal protein toxin
C14	1st and 2nd instar	Exposed to Cry3Bb1 maize Event MON88017 ^B^	Insecticidal protein toxin
C15	1st and 2nd instar	Exposed to Gpp34/Tpp35Ab1 maize Event DAS-59122-7 ^C^	Insecticidal protein toxin
C16	1st and 2nd instar	Exposed to drought conditions	Weather stress
C17	2nd and 3rd instar	Exposed to *Heterorhabditis bacteriophora*	Entomopathogen (nematode)
C18	2nd and 3rd instar	Exposed to *Metarhizium anisopliae*	Entomopathogen (fungus)
C19	Adult	Exposed to Thiomethoxam insecticide	Chemical insecticide
C20	Adult	Exposed to Curcurbitacin feeding stimulant	Semiochemical attractant

A. Syngenta, Basel, Switzerland;

B. Bayer Crop Sciences/Monanto Company, St. Louis, MO, USA

C. Corteva/Pioneer, Indianapolis, IN, USA

**Table 2:** Treatments performed in triplicate on *Diabrotica virgifera virgifera* inbred line Ped12 prior to RNA-seq library construction and sequencing

ID	Treatment	Treatment (exposure) description	Libraries (SRA Accessions)
T1	DiapEgg	Eggs diapause conditions.	LibA, LibB, LibC (ERR2791371–27913713)
T2	1st-Instar	1st instars.	LibD, LibE, LibF (ERR2791374–2791376)
T3	Ma	2nd and 3rd instars exposed to *Metarhizium anisopliae* (Ma).	LibG, LibH, LibI (ERR2791378–2791380)
T4	Gut	3rd instars exposed to the non-Bt hybrid Pioneer 38B85; fore-, mid- and hindgut	LibJ, LibK, LibL (ERR2791381–2791383)
T5	Hx	3rd instars exposed to maize Event DAS-59122-7; Herculex® XTRA Mycogen hybrid 2 T789 (Gpp34/Tpp35Ab1) for 2 days.	LibM, LibN, LibO (ERR-2791384-2,791,386)
T6	Hb	2nd and 3rd instars infected with *Heterorhabditis bacteriophora* (Hb).	LibP, LibQ, LibR (ERR2791387–2791389)
T7	Cn	3rd instars exposed to non-Bt hybrid 38B85 (control).	LibS, LibT, LibU (ERR2791390–2791392)
T8	VT3	3rd instars exposed to Event MON88017; DeKalb VT TriplePro® hybrid (Cry3Bb1) for 2 days.	LibV, LibW, LibX (ERR2791393–2791395)

Library read data used for estimates of differential gene expression are highlighted gray. All experimental accessions are located under BioSample ERS2715388; BioProject PRJEB28633

### Complementary DNA libraries, sequencing and data processing

**Fig. 1:**
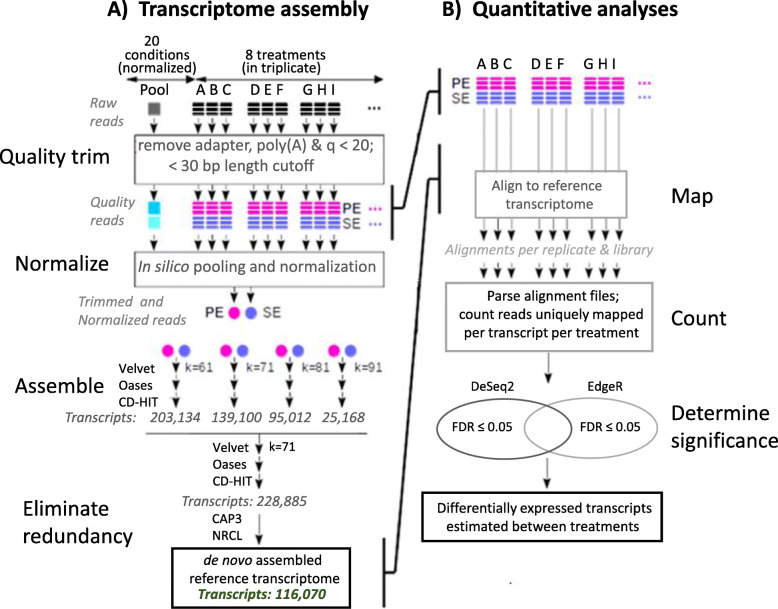
Transcriptome assembly and gene expression pipelines. Strategies for **A)** de novo assembly of the reference *Diabrotica virgifera virgifera* transcriptome, and **B)** estimation of mRNA quantities (using library read counts) and determination of statistical significance of any differences between treatments

A total of 769.6 million reads were obtained after trimming, and nearly 600 million (78%) remained PE reads (Table 3a). Among all reads, 249.7 million (97.4 paired and 55.0 singletons) were produced from the normalized Pooled library (Supplementary Table S1). 21.7 ± 8.5 (mean + SE; range: 8.3 to 34.2) million reads were generated from among replicates of the non-normalized RNA-seq libraries. All resulting trimmed reads were used in the construction of the *D. v. virgifera* reference transcriptome, and the non-normalized libraries were used to estimate read counts as a proxy for predicting differentially expressed transcripts (Fig. 1). All raw Illumina read data were submitted to GenBank Sequence Read Archive (SRA) database under accessions ERR2791371 to ERR2791395 (Table 2; BioProject PRJEB28633; BioSample SAMEA4896309).

**Table 3:** Representation of the de novo assembled *Diabrotica virgifera virgifera* reference transcriptome

A) Assembly and predictions	
Number of trimmed reads	769,834,956
Paired end reads	598,089,648
Single end reads	171,745,308
Number of in silico normalized reads	82,501,989
Paired end reads	51,579,260
Single end reads	30,922,729
Total assembled transcripts (≥ 200 bp)	228,885
Non-redundant assembled transcripts*	116,070
Minimum transcript length (nt)	200
Maximum transcript length (nt)	33,331
Total assembly size (bases)	101,481,135
N50	1197
Mean transcript size (nt)	875
Transcripts with predicted peptide	56,656
Predicted peptides with PFAM annotation	23,728
**B) Comparative analyses**	
Transcripts with a BLASTx hit**	
SwissProt database (v2015-02-04)	20,961
*Drosophila melanogaster* peptides v5.46	17,322
*Tribolium castaneum* proteins v3.0	24,543
*Dendroctonus ponderosae* proteins	26,965
*A. glabripennis* proteins	25,340
Overall^+^ covering ≥80% of subject length	** 14,574**
Overall^+^ putative full-length	** 7568**

* after redundancy suppression;

** *E*-value threshold = 10^−7^ and HSP cut-off = 25 (AA);

+ independent and non-overlapping set

### De novo reference transcriptome assembly and annotation

The *D. v. virgifera* reference transcriptome was assembled from all trimmed reads from the normalized cDNA library conditions (C1 to C20: Table 1) and non-normalized RNA-seq treatments (T1 to T8: Table 2), that used 82.5 million in silico normalized sequences (Table 3a). The multiple k-mer assembly approach was used (k = 61, 71, 81, 91) to generate a total of 228,885 redundant transcripts (Fig. 1a). After reduction in sequence redundancy and implementing a size cutoff ≥200 bp, a total of 116,070 transcripts comprised the final *D. v. virgifera* reference transcriptome (Fig. 1a; Table 3a), and were submitted to the NCBI Transcript Sequence Assembly (TSA) database (accession ERZ1775117.1).

Completeness was estimate by the presence of Core Eukaryotic Genes Mapping Approach (CEGMA) genes and BUSCO scores. Among the 248 CEGs, 98% were present in *D. v. virgifera* reference transcriptome (*n* = 243; mean 2.16 transcripts per CEG; 5 CEGs present as partial transcripts). A BUSCO score of 91.7% was obtained: 978 complete (821 complete single copy and 157 complete duplicated), 35 fragmented, and 53 missing BUSCOs were predicted.

Protein coding sequences were predicted within 56,656 of the 116,070 non-redundant *D. v. virgifera* transcripts, of which 23,728 received PFAM annotations (Table 3a). Among the 116,070 transcripts, 410 (0.35%), 107 (0.09%), and 65 (0.06%) were predicted to be putative *Wolbachia*, *Heterorhabditis* and *Metarhizium* transcripts, respectively (remaining data not shown). BLASTx searches resulted in matches to protein models from *D. melanogaster* (*n* = 17,322), *T. castaneum* (*n* = 24,543), *D. ponderosae* (*n* = 26,965), and *A. glabripennis* (*n* = 25,340), as well as in the SwissProt database (*n* = 20,961) (Table 3b; Supplementary Fig. S1). These results predicted that 7568 distinct *D. v. virgifera* transcripts to be “putative full-length” (proportional coverage lengths = 1.0) and 14,574 were “near complete (proportional coverage lengths ≥ 0.8 and <  1.0; Table 3b).

### Estimates of quantitative differences in transcript expression

**Fig. 2:**
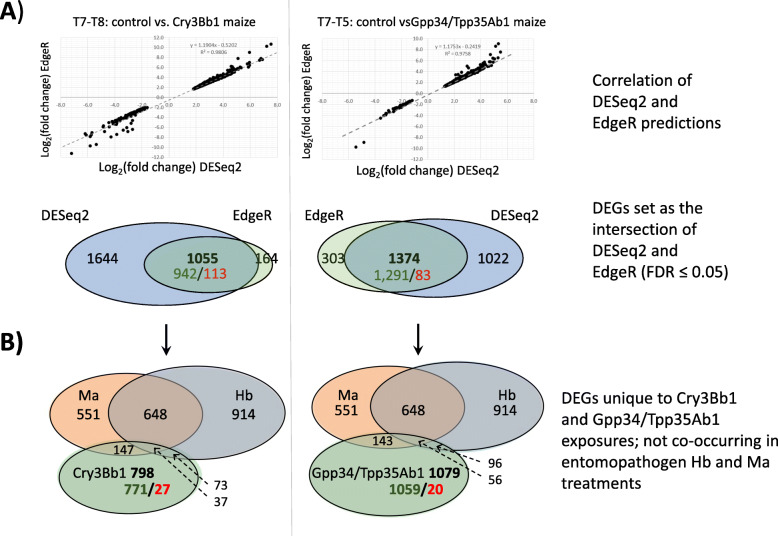
Prediction and filtering of putatively differentially expressed transcripts among susceptible *Diabrotica virgifera virgifera* larvae exposed to maize roots expressing *Bacillus thuringiensis* (Bt) pesticidal proteins. **A)** Correlation between Log_2_(fold-change) estimates between DESeq2 and EdgeR statistical packages for predicted differentially-expressed transcripts among larvae exposed roots that express Cry3Bb1 (T8; VT TriplePro® (VT3) hybrid) or Gpp34/Tpp35Ab1 (T5; Herculex® XTRA (Hx) hybrid) compared to non-Bt control maize (T7; Cn). Venn diagrams indicate the number of predicted differentially-expressed transcripts by DESeq2 and EdgeR surpassing significance thresholds (FDR ≤ 0.05), where the intersections (green up-regulated; red down-regulated) indicate those significant within both analyses. **B)** Comparison of differentially expressed transcripts between Bt and entomopathogen treatments, *Heterorhabditis bacteriophora* (Hb) and *Metarhizium anisopliae* (Ma). Differentially expressed transcripts within the intersection of Bt and entomopathogen treatments were subtracted to arrive at final filtered sets for Cry3Bb1 (T8; VT3) and Gpp34/Tpp35Ab1 (T5; Hx) maize treatments (green up-regulated; red down-regulated)

A total of 520.2 million reads (86.3%) were aligned to transcripts within the *D. v. virgifera* reference transcriptome (65.0 ± 21.5 million across treatments; 21.7 ± 8.5 million across replicates within treatments). Reads with multiple alignments, and those for which the mate-pair was aligned to a different target were discarded. Approximately half (52.1%) mapped properly (range 0.4036 and 0.5906; Supplementary Table S2). From these alignments, estimates of significant differences in read count (proxy for gene expression) for each transcript were generated from among replicates between control maize (Cn) relative to exposure treatments Cry3Bb1 (VT3; T8; Supplementary Table S3) and Gpp34/Tpp35Ab1 (Hx; T5; Supplementary Table S4), as well as entomopathogens *H. bacteriophora* (Hb; T6; Supplementary Table S5) and *M*. *anisopliae* (Ma; T3; Supplementary Table S6). Data from the comparison between T7 (control maize; Cn) and T8 (Cry3Bb1 maize; VT3) treatment groups, produced a DESeq2 adjusted read count for each transcript fitted to a dispersion around an empirically estimated mean (Supplementary Fig. 2A) and this was used to determine the significance of differences between treatments. Outliers within this dispersion were not fitted and not used in further DESeq2 analyses. MA-plots of estimated mean read counts normalized by size factor and transformed on a Log_2_(fold-change) scale showed that 2710 transcripts had differences in quantity that surpassed a Benjamini and Hochberg adjusted significance threshold (FDR) of ≥ 0.05. Among these transcripts, a greater number were up-regulated (*n* = 2503) than down-regulated (*n* = 207; Supplementary Fig. S2B; Supplementary Table S3). EdgeR estimated 1228 differentially-expressed transcripts for the same comparison between T7 and T8 with a greater number up-regulated (*n* = 1014) than down-regulated (*n* = 214). There was a strong correlation between Log_2_(fold-change) estimates between DESeq2 and EdgeR methods (R^2^ = 0.9806; Fig. 2a).

The comparison of read count data between replicate libraries from control maize (Cn; T7) versus transgenic Gpp35/Tpp35Ab1 maize exposed larvae (Hx; T5) similarly resulted in adjusted dispersions fitted to the mean (Supplementary Fig. S2C), and a final distribution of Log_2_(fold-change) in an MA plot (Supplementary Fig. S2D). DESeq2 output predicted 2389 transcripts that surpassed an adjusted significance threshold (FDR ≤ 0.05; Supplementary Table S4). EdgeR analysis of the same data predicted 1690 differentially expressed transcripts. Overall estimates of Log_2_(fold-change) were highly correlated between EdgeR and DESeq2 (R^2^ = 0.9758; Fig. 2a). Significant levels of differential expression (FDR ≤ 0.05) were predicted between Cn and Hb treatments for 1818 and 2376 transcripts using DESeq2 and EdgeR, respectively (Supplementary Table S5). Similarly, significant levels of differential expression (FDR ≤ 0.05) were predicted between Cn and Ma treatments for 1583 and 1684 transcripts by DESeq2 and EdgeR, respectively (Supplementary Table S6**)**. No other comparisons were conducted or evaluated.

### Differential expression following Cry3Bb1 and Gpp34/Tpp35Ab1 exposure

Our pipeline defined differential expression occurring only among transcripts having an adjusted *P*-value (FDR) ≤ 0.05 in both DESeq2 and EdgeR analyses (Fig. 1**b**). Furthermore, any differentially expressed transcripts after entomopathogen exposures and pesticidal protein treatments were subtracted to account for putative general stress response genes. Specifically, 1562 transcripts showed significant differential expression among *D. v. virgifera* exposed to *H. bacteriophora* compared to control larvae in both DESeq2 and EdgeR analyses, of which 1269 and 293 were up- and down-regulated, respectively (Table 4; Supplementary Table S5). Analogously, 1199 differentially expressed transcripts were predicted between *M. anisopliae* exposure and control treatments, with 639 and 563 transcripts up- and down-regulated, respectively (Table 4; Supplementary Table S6). Among the most prevalent PFAM annotations assigned to predicted differentially-regulated transcripts in both Hb and Ma treatments were those with cytochrome P450, transporter, and protease and protease inhibitor domains (Supplementary Table S7; Supplementary Table S8).

**Table 4:** Count of differentially expressed transcripts between treatment pairs (FDR ≤ 0.05 in DESeq2 and EdgeR)

	ID/Treatment (as in Table 2)			
	T7-T8	T7-T5	T7-T3	T7-T6
Transcript partitions	Cn v. VT3	Cn s. Hx	Cn v. Ma	Cn v. Hb
Total differentially expressed	1064	1387	1199	1562
*total up-regulated*	950	1296	638	1269
*total down-regulated*	114	91	563	293
*total with PFAM domains*	773	1031	658	820
*total with signal peptide*	293	366	212	204
Endogenous* (total/Bt only)^*#*^	1055/ 798	1374/1079	NA	NA
*up-regulated (total/Bt only)*^*#*^	942/ 771	1291/1059	636/NA	1269/NA
*down-regulated (total/Bt only)*^*#*^	113/ 27	83/ 20	563/NA	293/NA

* no significant BLASTx hits to *Wolbachia*, *Heterorhabditis bacteriophora* (Hb)*, or Metarhizium anisopliae* (Ma) protein databases (*E*-values > 1.0^− 7^)

# Bt only values obtained by filtering differentially expressed transcripts from Cry3Bb1 or Gpp34/Tpp35Ab1 datasets which were also differentially expressed in Hb and Ma treatments

NA Not applicable

**Fig. 3:**
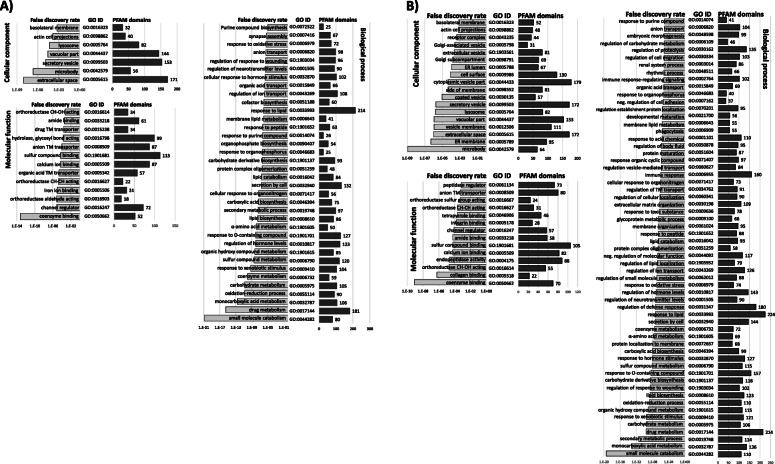
Enriched Gene Ontology (GO) terms among transcripts differentially expressed in susceptible *Diabrotica virgifera virgifera* larvae exposed to maize roots expressing *Bacillus thuringiensis* pesticidal proteins**.** For independent **A)** Cry3Bb1* and **B)** Gpp34/Tpp35Ab1 treatments* significantly over-represented GO terms are shown within the categories of biological process (BP) and cellular component (CC) (FDR ≤ 1.0E^− 5^), and molecular function (MF) at level 2 (FDR ≤ 1.0E^− 6^; gray bars). Categories listed by GO ID and GO term in order of highest significance (bottom) to lowest significance (top). Number of transcripts encoding each PFAM domain within each functional category are indicated (black bars). * Filtered differentially expressed transcripts following in silico subtraction that removed those shared with transcripts also differentially expressed within entomopathogen treatments (Hb and Ma)

Comparison between T7 (control maize; Cn) and T8 (Cry3Bb1 maize; VT3) treatments predicted a total of 1064 differentially expressed transcripts by both DESeq2 and EdgeR (Table 4; Supplementary Table S3). Subsequent BLASTx results showed 4 and 5 of these transcripts putatively originated from *Wolbachia* sp., and the entomopathogens Hb and Ma, respectively. Among the remaining 1055 transcripts 942 and 113 were up- and down-regulated, respectively (Table 4; Fig. 2a). Comparison showed that 257 endogenous transcripts differentially expressed between Cry3Bb1 and controls were also differentially expressed in Hb and/or Ma treatments compared to controls (Fig. 2b). GO enrichment analyses of these shared transcripts predicted secretory vesicle (category CC), C-N bonding forming ligase activity (MF), and purine compound biosynthesis process (BP) among the most significantly overrepresented (Supplementary Fig. S3A). After removal of these 257 transcripts shared with Hb and Ma treatments 798 endogenous transcripts were unique to the Cry3Bb1 response. Of the 775 unique PFAM domain annotations assigned to 609 of these 798 differentially expressed transcripts (76.3%), cathepsin inhibitor (Inhibitor_I29), papain (Peptidase_C1) and trypsin proteases functional domains were most prevalent (Table 5). Transcripts encoding alkaline phosphatase, aminopeptidase, or cadherin domains were not among those that were differentially expressed. Annotations did indicate that three transcripts up-regulated in Cry3Bb1 treatments encoded putative ABC transporter-like proteins from subfamilies ABCG (*n* = 1) and ABCC (*n* = 2) (Table 6), while a single transcript was annotated with a tetraspanin domain (DIAVI021979). Transcripts putatively encoding six apoptosis-related proteins, including two caspases, one IAP, and the BAX-domain containing protein BI-1 were up-regulated by Cry3Bb1 exposed larvae (Table 7). The most significantly enriched GO terms assigned at level 2 to differentially expressed transcripts in the Cry3Bb1 treatment were in extracellular space and microbody (category CC), coenzyme binding and channel regulator activities (MF), and small molecule catabolism and drug metabolism processes (BP) (Fig. 3a).

**Table 5:** Predicted PFAM domains encoded by transcripts differentially-expressed in susceptible *Diabrotica virgifera virgifera* Ped12

				Comparisons		
PFAM domain name				T7-T5	T7-T8	Both
	PFAM domain description	PFAM_ID	InterPro_ID	Hx v Cn	VT3 v Cn	∩
Sugar_tr	Sugar transporter	PF00083	IPR005828	26	17	14
p450	Cytochrome P450	PF00067	IPR001128	22	17	15
Lectin_C	Lectin C-type domain	PF00059	IPR001304	21	12	11
Inhibitor_I29	Cathepsin propeptide inhibitor domain	PF08246	IPR013201	19	22	14
Trypsin	Trypsin	PF00089	IPR001254	19	20	9
Peptidase_C1	Papain family cysteine protease	PF00112	IPR000668	18	21	13
CBM_14	Carbohydrate binding module	PF01607	IPR002557	17	15	12
LRR_8	Leucine-rich repeat	PF13855	IPR001611	15	11	10
COesterase	Carboxyesterase, type B	PF00135	IPR002018	14	18	10
adh_short	Short chain dehydrogenase	PF00106	IPR002347	13	5	3
EcKinase	Ecdysteroid kinase	PF02958	IPR004119	11	12	8
UDPGT	UDP glycosyltransferases	PF00201	IPR002213	11	11	8
Kunitz_BPTI	Kunitz/Bovine pancreatic	PF00014	IPR002223	10	3	3
MFS_1	Major facilitator superfamily	PF07690	IPR011701	10	10	7
DUF1397	Unknown function	PF11901	IPR024518	9	3	3
Serpin	Serpin serine protease inhibitor	PF00079	IPR026796	9	3	3
Abhydrolase_1	Alpha/beta hydrolase fold	PF00561	IPR000073	8	11	6
Glyco_hydro_18	Glycosyl hydrolase family 18	PF00704	IPR001223	7	5	4
LRR_5	BspA type Leucine rich repeat region	PF13306	IPR026906	7	4	4
EF-hand_7	EF-hand domain pair	PF13499	IPR002048	6	3	3
Fibrinogen_C	Fibronectin	PF00147	IPR002181	6	7	4
GST_C	Glutathione S-transferase, C-terminal	PF00043	IPR004046	6	7	5
PBP_GOBP	PBP/GOBP family	PF01395	IPR006170	6	2	2
ADH_zinc_N	Zinc-binding dehydrogenase	PF00107	IPR013149	5	3	2
Aldo_ket_red	Aldo/keto reductase	PF00248	IPR023210	5	6	3
Ank_2	Ankyrin repeats	PF12796	IPR020683	5	4	3
Far-17a_AIG1	Far-17a_AIG1-like protein	PF04750	IPR006838	5	4	4
GILT	γ-interferon-inducible lysosomal thiol	PF03227	IPR004911	5	5	4
Glyco_hydro_1	Glycosyl hydrolase family 1	PF00232	IPR001360	5	14	5
Ig_2	Immunoglobulin domain	PF13895	IPR007110	5	0	0
JHBP	Juvenile hormone-binding protein	PF06585	IPR010562	5	10	4
Lipase	Lipase	PF00151	IPR013818	5	4	3
Ras	Ras family	PF00071	IPR001806	5	3	3
7tm_2	7 transmembrane receptor	PF00002	IPR000832	4	4	4
ABC2_membrane	ABC-2 type transporter	PF01061	IPR013525	4	2	1
ABC_tran	ABC transporter	PF00005	IPR003439	4	2	1
Abhydro_lipase	Alpha/beta hydrolase lipase region	PF04083	IPR006693	4	6	2
Asp	Eukaryotic aspartyl protease	PF00026	IPR033121	4	3	2
DUF3421	Protein of unknown function	PF11901	IPR024518	4	4	3
GST_N	Glutathione S-transferase, N-terminal	PF02798	IPR004045	4	5	3
I-set	Immunoglobulin I-set domain	PF07679	IPR013098	4	4	3
Lys	C-type lysosome/alpha-lactalbumin	PF00062	IPR001916	4	0	0
Sina	Seven in abstensia protein family	PF03145	IPR018121	4	4	3
SVWC	von Willebrand factor type C	PF15430	IPR029277	4	4	4
Tetraspannin	Tetraspannin family	PF00335	IPR018499	4	2	2

Counts predicted within translated products for differentially-expressed transcripts for Bt susceptible *D. v. virgifera* Ped12 larval exposures to transgenic maize roots that express Gpp34/Tpp35Ab1 (T5; Herculex® (Hx) hybrid) and Cry3Bb1 (T8; VT TriplePro® (VT3) hybrid) compared to control (T7). The intersection of differentially-expressed transcripts in both Cry3Bb1 and Gpp34/Tpp35Ab1 treatments also indicated. All counts representative of transcripts after in silico subtraction of those also differentially-expressed in exposures to the entomopathogens *Heterorhabditis bacteriophora* (Hb) and *Metarhizium anisopliae* (Ma). Values provided for instances when ≥4 transcripts received an annotation within at least one of the treatments. InterPro identification (InterPro_ID) are also given

**Table 6:** Candidate *Bacillus thuringiensis* (Bt) insecticidal protein receptors differentially in susceptible *Diabrotica virgifera virgifera* Ped12 larvae

			Log_2_(fold-change)				
		Putative	Bt maize		Entmopathogens		*Tribolium castaneum* ortholog
Transcript	Dvir_v2.0 Protein model(s)#	annotation	T7-T8	T7-T5	T7-T6	T7-T3	(Assn; GLEAN; Ortholog; ID/Aligned L)
DIAVI009217	XP_028139024.1 & 25.1	ABC subfamily G	+ 2.42	NS	NS	NS	XP_973526.1; 07047; *TcABCG-4H*; 53%/ 646
DIAVI012990	XP_028134948.1 to 52.1	ABC subfamily C	+ 2.41	+ 2.28	NS	NS	XP_969781.1; 14,775; *TcABCC-ST*; 57%/ 439
DIAVI001032	XP_028145606.1	ABC subfamily C	+ 2.07	NS	NS	NS	XP_8193834.1; 14,403; *TcABCC-SR*; 44%/1374
DIAVI006431	XP_028139807.1	ABC subfamily B	NS	+ 1.98	NS	NS	XP_974441.2; 15,192; *TcABCB-6A*; 77%/ 612
DIAVI025188	XP_028135097.1	ABC subfamily G	NS	+ 1.96	NS	NS	XP_008198312.1; ND; ND; 36%/ 156
DIAVI008609	XP_028129515.1 & 16.1	ABC subfamily E	NS	+ 1.58	NS	NS	XP_968009.1; 10,519; *TcABCE-3A*; 91%/ 557
DIAVI001096	XP_028128982.1	ABC subfamily C	NS	NS	+ 0.81	NS	XP_972534.1; 09892; *TcABCC-7B;* 63%/1280
DIAVI001196	XP_028139572.1	ABC subfamily C	NS	NS	+ 2.39	+ 2.13	XP_971802.2; 14,383; *TcABCC-5 N;* 44%/1260
DIAVI001308	XP_028155748.1	ABC subfamily C	NS	NS	+ 1.57	NS	XP_971908.1; 14,385; *TcABCC-5P*; 46%/1266
DIAVI001589	XP_028142713.1	ABC subfamily C	NS	NS	+ 1.16	+ 1.03	XP_971802.2; 14,383; *TcABCC-5 N; 45%/1280*
DIAVI001763	XP_028131534.1	ABC subfamily C	NS	NS	+ 1.11	+ 1.22	XP_971802.2; 14,383; *TcABCC-5 N; 48%/1258*
DIAVI001938	XP_028155747.1	ABC subfamily C	NS	NS	−1.38	−2.05	XP_971908.1; 14,385; *TcABCC-5P*; 47%/1240
DIAVI012237	XP_028137191.1 to 93.1	ABC subfamily C	NS	NS	NS	−2.17	XP_971802.2; 14,383; *TcABCC-5 N; 47%/ 587*
DIAVI021979	XP_028140422.1	Tetraspanin	+ 2.48	+ 2.34	NS	NS	XP_969428.111703; CD63 antigen;53%/ 271
DIAVI004770	XP_028137187.1 & 88.1	Tetraspanin	NS	+ 1.84	NS	NS	XP_966752.212071; *Tetraspanin33*: 91%; 291

Fold-change and direction (+ or -) of differential expression among transcripts is indicated within exposures to roots of maize hybrids that express Bt pesticidal proteins Cry3Bb1 (T8; VT TriplePro® (VT3) hybrid) and Gpp34/Tpp35Ab1(T5; Herculex® (Hx) hybrid), or entomopathogens *Heterorhabditis bacteriophora* (T6) and *Metarhizium anisopliae* (T3), compared to the non-Bt maize control treatment (T7)

# Protein models in RefSeq accession GCF_003013835.1 annotated from assembly accession GCA_003013835.2 under BioProject PRJNA432972; NS: not significant with FDR > 0.05; ND: not determined; Accn: GenBank accession; GLEAN: *Tribolium castaneum* GLEAN protein nomenclature; Ortholog: *T. castaneum.* ABC transporter classification according to Broehan et al. (2013) and putative annotation as defined by Adepipe et al. (2019); ID: identity from BLASTp alignments; Aligned L: length of BLASTp alignment

**Table 7:** Differential expressed apoptotic and autophagy-related protein-encoding transcripts

		Log_2_(fold-change)			
Transcript	Putative annotation	T7-T8	T7-T5	T7-T6	T7-T3
DIAVI022989	Initiator caspase-like (orthologs: STRICA or DAMM)	+ 3.21	+ 3.05	ns	ns
DIAVI027204	Effector caspase-like (ortholog: DECAY)	+ 3.00	+ 2.47	ns	ns
DIAVI057195	Stress-associated endoplasmic reticulum protein 2	+ 2.26	+ 2.13	ns	ns
DIAVI029891	Lifeguard 4-like (BAX inhibitor domain protein)	+ 2.02	+ 1.76	ns	ns
DIAVI013501	Autophagy-related gene 13 (atg13)-encoded protein	+ 2.20	+ 1.71	−1.42	−1.52
DIAVI011561	Programmed cell death protein 4	ns	+ 1.67	ns	ns
DIAVI026079	BAX inhibitor 1 (BI-1)	ns	+ 1.52	ns	ns
DIAVI011972	Inhibitor of apoptosis 1 (IAP1) isoform X1	ns	+ 2.22	ns	ns
DIAVI007715	Inhibitor of apoptosis 1 (IAP1) isoform X5	+ 4.36	ns	ns	ns

Response among susceptible *Diabrotica virgifera virgifera* Ped12 larvae exposed to *Bacillus thuringiensis* (Bt) maize roots and entomopathogens. Fold-change and direction (+ or -) of differential expression for transcripts is indicated for treatments with exposures to maize roots that express Bt pesticidal proteins Cry3Bb1 (T8; VT TriplePro® (VT3) hybrid)) and Gpp34/Tpp35Ab1 (T5; Herculex® (Hx) hybrid), and to the entomopathogens *Heterorhabditis bacteriophora* (T6) and *Metarhizium anisopliae* (T3), compared to non-Bt control (T7). Transcripts differentially expressed in the intersection of both Bt maize treatments are highlighted in gray. # Putative homologs from protein models in RefSeq accession GCF_003013835.1 annotated from genome assembly accession GCA_003013835.2 under the BioProject PRJNA432972: DIAVI022989 = XP_028132548.1; DIAVI027204 = XP_028144295.1; DIAVI057195 = XP_028135864.1; DIAVI029891 = XP_028155171.1; DIAVI013501 = XP_028130453.1; DIAVI011561 = XP_028128855.1 and XP_028128856.1; DIAVI026079 = XP_028141533.1; DIAVI011972 and DIAVI007715 = XP_028141650.1 to 58.1

Comparisons between control (Cn; T7) and Gpp35/Tpp35Ab1 maize (Hx; T5) treatments predicted significant differences for 1387 transcripts by both DESeq2 and EdgeR (Table 4; Supplementary Table S4). Among these transcripts, 13 showed top BLASTx hits to *Wolbachia* sp. (*n* = 7), *H. bacteriophora* (*n* = 1), or other microbial protein database sources (*n* = 5), and were removed from the dataset. This filtered set of 1374 *D. v. virgifera* transcripts (Table 4**;** Fig. 2a) contained 295 that were also differentially expressed in one or both of the Hb and/or Ma treatments (Fig. 2b). GO enrichment analyses predicted the most significant over-representation was for secretory vesicle (category CC), coenzyme binding activity (MF), and organophosphate biosynthesis process (BP) (Supplementary Fig. S3B). Following removal of these 295 transcripts shared with Hb and Ma responses, a total of 1079 were retained in the dataset of endogenous transcripts uniquely responding to Gpp34/Tpp35Ab1 (Fig. 2b). A total of 1066 PFAM domain annotations were assigned to 837 of these 1079 differentially-expressed transcripts (77.6%), with sugar transporter (Sugar_tr), cytochrome P450 (p450), and lectin C-type domain (Lectin_C) numerically greatest (Table 5). No alkaline phosphatase, aminopeptidase, or cadherin domains were annotated among differentially expressed transcripts. PFAM domains for ABC transporter were assigned to four up-regulated transcripts, one assigned to each of the ABCB, C, G, and E subfamilies (Table 6). By comparison, a total of 6 and 5 transcripts encoding ABCC subfamily members were differentially regulated in *H. bacteriophora* and *M. anisopliae* treatments, respectively (Table 6), but none were predicted in common with those in the Cry3Bb1 or Gpp34/Tpp35Ab1 treatments. A set of nine apoptosis-related protein-encoding transcripts were up-regulated in Gpp34/Tpp35Ab1 exposed larvae, of which five were in common with those also up-regulated in Cry3Bb1 maize (Table 7). Transcripts uniquely up-regulated following exposure to Gpp34/Tpp35Ab1 maize included one putatively encoding a BI-1 ortholog, and a different IAP1 isoform (X1) than the IAP1 isoform X5 up-regulated in Cry3Bb1 treatments. Enrichment at GO level 2 showed greatest significance within in microbody and secretory vesicle (category CC), coenzyme binding activity (MF), and small molecule catabolism processes (BP) in the Gpp34/Tpp35Ab1 treatment (Fig. 3b).

**Fig. 4:**
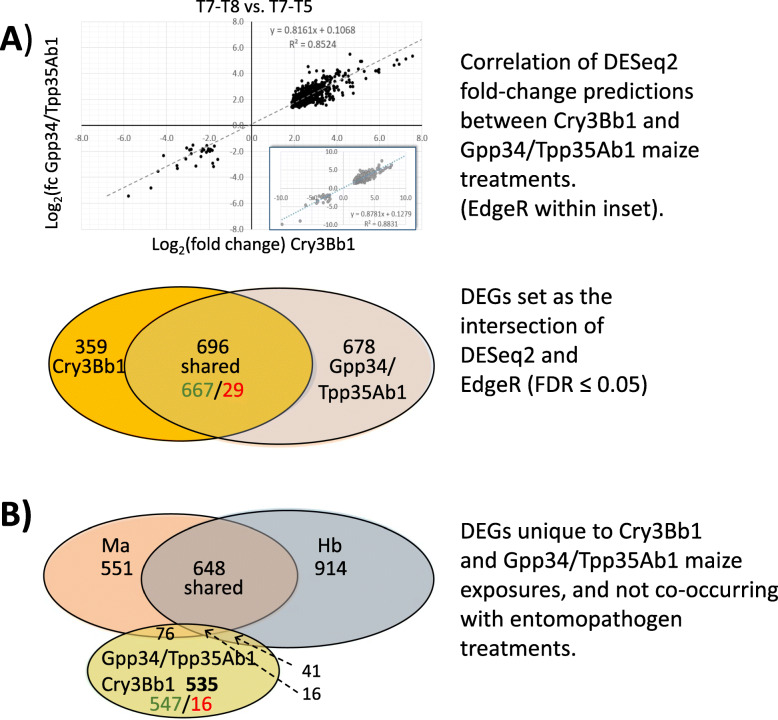
Prediction and filtering of the intersection of transcripts putatively differentially expressed among susceptible *Diabrotica virgifera virgifera* larvae exposed within independent treatments to maize roots expressing different *Bacillus thuringiensis* (Bt) pesticidal proteins. **A)** Correlation between estimated Log_2_(fold-change) among differentially-expressed transcripts from DESeq2 and EdgeR (FDR ≤ 0.05) between larvae exposed to non-Bt control hybrid maize roots (T7) with maize roots that express Bt Cry3Bb1 (T8; VT TriplePro® (VT3) hybrid) and Gpp34/Tpp35Ab1 (T5; Herculex® (Hx) hybrid). Venn diagram indicates the number of predicted differentially-expressed transcripts by DESeq2 and EdgeR surpassing significance thresholds (FDR ≤ 0.05), where the intersections (green up-regulated; red down-regulated) indicate those significant within both analyses. **B)** Comparison of differentially expressed transcripts between Bt and entomopathogen treatments, *Heterorhabditis bacteriophora* (Hb) and *Metarhizium anisopliae* (Ma). Differentially expressed transcripts within the intersection of those shared by both Bt and entomopathogen treatments, that were subtracted to arrive at final filtered set shared transcripts uniquely differentially expressed in both Cry3Bb1 (T8; VT3) and Gpp34/Tpp35Ab1 (T5; Hx) maize root treatments (green up-regulated; red down-regulated)

**Fig. 5:**
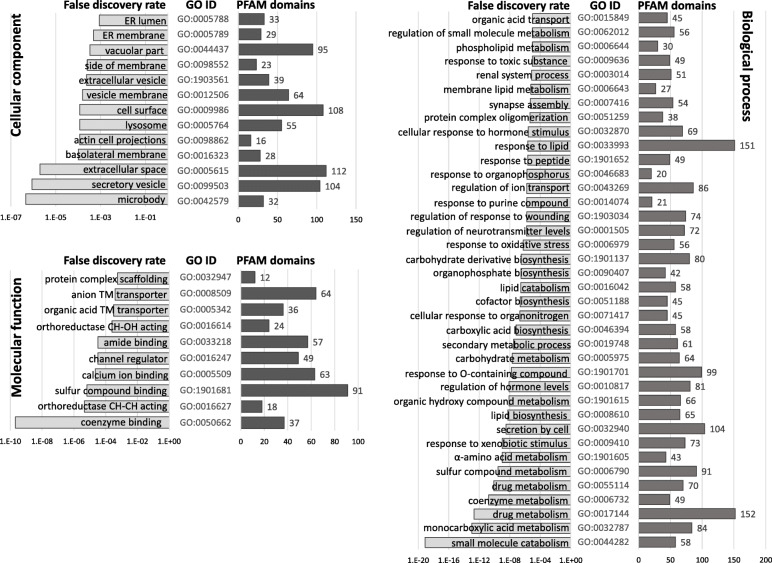
Enriched Gene Ontology (GO) terms among transcripts within the intersection of differentially expressed in susceptible *Diabrotica virgifera virgifera* larvae independently exposed to maize roots expressing different *Bacillus thuringiensis* (Bt) pesticidal proteins*. Significantly over-represented GO terms within the categories of biological process (BP), cellular component (CC) (FDR ≤ 1.0E^− 5^), and molecular function (MF) at level 2 (FDR ≤ 1.0E^− 6^; gray bars). Categories listed by GO ID and GO term in order of highest significance (bottom) to lowest significance (top). Number of transcripts encoding each PFAM domain within each functional category are indicated (black bars). * Set of 563 transcripts with estimated significant levels of differential expression between *D. v. virgifera* larvae exposed to hybrid maize roots that express Bt Cry3Bb1 (T8; Hx) and Gpp34/Tpp35Ab1 (T5; Hx) compared to non-Bt control maize roots (T7; Cn) within independent treatments (intersection). These shared transcripts were further filtered by in silico subtraction to remove differentially expressed transcripts that were also differentially expressed within entomopathogen treatments (*Heterorhabditis bacteriophora* (Hb) and *Metarhizium anisopliae* (Ma))

A set of 696 differentially-expressed endogenous transcripts were shared between both Cry3Bb1 and Gpp34/Tpp35Ab1 treatments (Supplementary Table S9; Fig. 4a). The Log_2_(fold-change) estimates for these transcripts were highly correlated between DESeq2 and EdgeR analyses (R^2^ ≥ 0.8524; Fig. 4a). Following removal of 133 transcripts that were also differentially expressed in Hb and/or Ma treatments, a set of 535 endogenous transcripts were shared and responding to both Cry3Bb1 and Gpp34/Tpp35Ab1 (Supplementary Table S9; Fig. 4b). Predicted PFAM domains showed that of putative cytochrome P450 (p450), cathepsin inhibitor (Inhibitor_I29), sugar transporter (Sugar_tr), papain cysteine protease (Peptidase_C1), and carbohydrate binding module (CBM_14) were the five most prevalent (Table 5). GO enrichment at level 2 for differentially expressed transcripts shared in pesticidal protein exposures was greatest within CC categories microbody, secretory vesicle, and extracellular space (Fig. 5). Additionally, enrichment was greatest for coenzyme binding activity (MF), and small molecule catabolism, monocarboxylic acid metabolism, and drug metabolism processes (BP) (Fig. 5). An ABCC subfamily member putatively orthologous to the *T. castaneum* ABCC-ST gene was significantly up-regulated in the Cry3Bb1 and Gpp34/Tpp35Ab1 treatment (Table 6). Five apoptosis-related proteins were up-regulated in both Cry3Bb1 and Gpp34/Tpp35Ab1 treatments (Table 7). The transcript DIAVI004770, encoding a putative tetraspanin-like protein, was up-regulated in both Cry3Bb1 and Gpp34/Tpp35Ab1 exposure treatments.

Overall, these results show that after filtering, differentially-expressed transcripts in separate Cry3Bb1 and Gpp34/Tpp35Ab1 treatments encode proteins putatively most enriched for those localized in the extracellular space, secretory vesicles, and microbodies, and having coenzyme binding function and are involved in small molecule catabolism (Fig. 3). These same categories are also most significantly enriched among differentially expressed transcripts share in both treatments (Fig. 5). Of the putative Bt receptor proteins, only two were differentially expressed across Bt maize exposure larvae (Table 6), whereas five transcripts putatively encoding apoptosis and cell stress-related proteins were upregulated in both Cry3Bb1 and Gpp34/Tpp35Ab1 treatments (Table 7).

### Phylogenetics and structural annotations

**Fig. 6:**
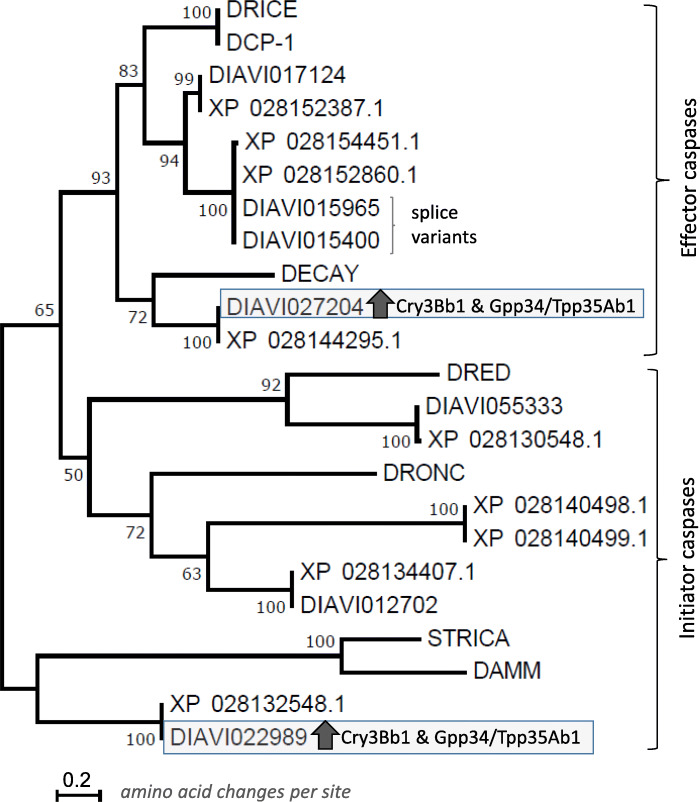
Phylogenetic relationship and orthology of putative *Diabrotica virgifera virgifera* caspases. Maximum-likelihood (ML) analysis of a partial enzymatic domain alignment between putative *D. v. virgifera* caspases with seven known caspases from *Drosophila melanogaster* (Supplementary Fig. 5). The *D. v. virgifera* transcripts (DIAVI0NNNNN) and protein models (XP 0281NNNNN.1) from RefSeq accession GCF_003013835.1 for the draft genome assembly accession GCF_003013835.1 Dvir_v2 are integrated for homolog estimation. Caspase-encoding transcripts DIAVI027204 and DIAVI022989 up-regulated in both Cry3Bb1 and Cry34/35Ab1 exposed larvae (Table 7) are enclosed in boxes. Clades corresponding to effector and initiator classes are indicated by brackets. The ML of the constructed tree using the LG model of protein sequence evolution (Le and Gascuel 2008) [77] with a gamma shape parameter (*G*) = 2.7553 and 6.77% of sites defined as evolutionarily invariable (*I*) was minimized at a log likelihood of − 2224.28, and resulted in a tree with a total branch length of 10.157. Node support obtained using 1000 bootstrap pseudo-replications of the aligned protein sequence data

BLASTp results showed that 7 *D. v. virgifera* transcripts (this study) and 9 *D. v. virgifera* gene models surpassed *E*-value thresholds against *D. melanogaster* caspases: DRONC, death regulator Nedd2-like caspase (FlyBase ID: FBgn0026404); DRED, Death related ced-3/Nedd2-like caspase (FBgn0020381), DAMM (FBgn0033659), STRICA, Ser/Thr-rich caspase (FBgn0033051), DECAY, Death executioner caspase related to Apopain/Yama (FBgn0028381), DCP-1, death caspase 1 (FBgn0010501), and DRICE, death related ICE-like caspase (FBgn001997; data not shown). A 197 amino acid consensus alignment was generated for a partial enzymatic domain region among seven *D. melanogaster* and putative *D. v. virgifera* caspases (Supplementary Fig. S4). Percent identity among aligned sequences ranged from 17.12 to 100.00, with catalytic histidine (H) and cysteine (C) residues 100% conserved. The LG + G + I model maximized the BIC score at 4496.352, and was implemented as the “Best Model” for subsequent phylogenetic reconstruction. The subsequent unrooted ML-based phylogeny had a G of 2.7553 and I of 6.77% that minimized at the log likelihood score of − 2224.28, resulting in a consensus tree of total branch length of 10.157 (Fig. 6). Two major clades comprised of effector and initiator caspases were supported by 65% of 1000 bootstrap pseudo-replicates of the data. Clade was defined based on phylogenetic position of *D. melanogaster* effector (DRICE, DCP-1 and DECAY) and initiator caspases (DRONC, DRED, STICA and DAMM), and predicted the effector DECAY and initiators STRICA or DAMM as nearest orthologs to up-regulated *D. v. virgifera* caspases DIAVI027204 and DIAVI022989, respectively (Fig. 6). Overall, each transcript within the assembled transcriptome showed a one-to-one phylogenetic correspondence with a Dvir_v2 protein model, with the exception of a lack of homologous transcripts for XP_028140498.1 and XP_028140499.1.

Local BLASTn and BLASTp searches of *D. v. virgifera* gene models using corresponding sequences from putative apoptosis- or cell stress-related proteins encoded by the differentially expressed transcripts, IAP, BI-1, and LFG, resulted in identification of nearest homologs. Queries of the CDD with proteins encoded by DIAVI007715 and DIAVI011972 differentially expressed following *D. v. virgifera* exposure to Cry3Bb1 or Gpp34/Tpp35Ab1, respectively, allowed classification of both as IAP family 1 (IAP1) members based on presence of two baculoviral inhibition of apoptosis protein repeat (BIR) domains (Supplementary Fig. S5A). BLASTn results indicated that DIAVI011972 and DIAVI007715 were derived from Dvir_v2.0 LOC114335598, and represent splice variants XM_028285849.1 (isoform X1; protein translation XP_028141650.1; *E*-value = 0.0, %ID = 100.0) and XM_028285854.1 (isoform X5; protein XP_028141655.1; *E*-value = 0.0, %ID = 100.0), respectively. By comparison, FPAM and CDD results predicted that the non-differentially expressed transcript DIAVI011430 (homolog of XP_028283750.1; Dvir_v2.0 LOC114333756 *E*-value = 0.0, %ID = 100.0), encoded three BIR domains which is structurally similar to *Drosophila* DIAP2 orthologs (Supplementary Fig. S5B). Alignments of *D. v. virgifera* IAP1 and IAP2 BIR domains with *D. melanogaster* orthologs DIAP1 and DIAP2 showed ≥ 22.39% and ≥ 26.47% identities, respectively (Supplementary Fig. S5C). Analogous results were obtained for BAX inhibitor-like, BI-1 (Supplementary Fig. S6A) and Lifeguard 4 (LFG4) (Supplementary Fig. S6B), and SERP2-like proteins (Supplementary Fig. S7).

These results showed that in Cry3Bb1 and Gpp34/Tpp34Ab1 treatments the two upregulated caspases belonged in two separate clades corresponding to initiator and effector caspases (Fig. 6), that function at different stages to propagate apoptosis-related proteolytic cascades. Transcripts upregulated in both treatments also included a IAP2 class protein that correspondingly repressed progression of caspase cascade, and the remaining upregulated transcripts BI-1, LGF4 and SERP2 show homology to related peptides with putative function in repression of cell death and promotion of cell survival.

## Discussion

### Transcriptome responses to Bt pesticidal protein exposures

The modes by which Bt intoxication evokes changes that elicit cell death or recovery, tissue damage or repair, and organismal mortality or survival are not yet fully understood [46, 66, 78]. Several studies demonstrate a role for gut membrane-bound protein receptors in mediating Bt intoxication, but uncertainties remain regarding molecular mechanisms underlying subsequent physiological and cellular changes. Specifically, pore-formation/osmotic imbalance, signal transduction, or hybrid models suggest different causes [27]. Studies comparing transcriptome-wide expression differenes between susceptible insects exposed or unexposed to Bt proteins can provide valuable mechanistic insights, but such studies tend to identify hundreds or thousand of differentially-expressed genes [74–76, 79–82]. This phenomenon is also observed as part of insect responses to chemical insecticide exposures [83] and pathogen infections [84]. Such outcomes not only impose challenges for interpreting results, but also in formulating hypotheses to guide future reseach.

In the current study we aimed to reduce the genetic component of variation in responses of *D. v. virgifera* by using an inbred strain Ped12. Additionally, we removed any transcripts from further consideration that were not identified by both DESeq2 and EdgeR packages, resulting in substantial reductions of 63.1 and 49.1% in candidate transcripts for Cry3Bb1 and Gpp34/Tpp35Ab1 exposures, respectively. Of the remaining transcripts, we performed in silico subtraction to remove transcripts that also were differentially expressed following exposures to entomopathogens (Hb or Ma), which was hypothesized to eliminate transcripts in shared general stress response pathways. These subtraction measures reduced the number of significantly differentially expressed transcripts in susceptible *D. v. virgifera* larvae exposed to transgenic Cry3Bb1 and Gpp34/Tpp35Ab1 maize to 798 and 1079, respectively (≥ 24.4% reduction) (Table 4**;** Fig. 2). Similarly, among the 696 differentially expressed transcripts shared between the two Bt maize treatments, an additional 133 (19.1%) were also differentially regulated in Hb and Ma treatments, leaving 563 after removal (Fig.4b). Among the filtered transcripts, are those encoding candidate Bt receptor proteins, metabolic and detoxification enzymes, and proteins putatively involved in cell fate (pro-survival or -death) were over represented, and these were focused upon in our further investigations.

### Up-regulation of putative *B. thuringiensis* receptors

Transcripts predicted to encode previously identified receptor proteins were differentially-expressed among susceptible *D. v. virgifera* exposed to Cry3Bb1 and/or Gpp34/Tpp35Ab1 compared to controls. For example, tetraspanin encoding transcripts were up-regulated in response to Cry3Bb1 (transcript DIAVI021979) and Gpp34/Tpp35Ab1 (transcripts DIAVI004770 and DIAVI021979; Table 6). A non-synonymous change in the transmembrane domain in the *H. armigera* tetraspanin gene, HaTSPAN1, was linked to Cry1Ac resistance in strain AY2 [51]. The HaTSPAN1 transcript levels are increased 2.7-fold in AY2, but did not alter Cry1Ac binding. The means by which structural changes and up-regulation of tetraspanin interrupts the Bt mode of action in *H. armigera* AY2 [51], or role of tetraspanin-like transcripts in Cry3Bb1 and Gpp34/Tpp35Ab1 responses by susceptible *D. v. virgifera* remains unknown. Alternate midgut receptors in resistant insects have been proposed to sequester Bt proteins, thereby reducing binding to membrane-bound proteins functionally involved in pore formation [85, 86], such as ABC transporters, cadherin, aminopeptidase N or alkaline phosphatases. Regardless, a potential protein sequestering role of tetraspanin remains to be investigated.

The involvement of ABC transporters in Bt intoxication has been demonstrated through linkage or association with resistance among species of Lepidoptera to subfamily members *abcc2* [34, 56, 57, 60], *abca2* [87], and *abcg* [88, 89]. In the current study of Bt susceptible *D. v. virgifera* larvae, five transcripts encoding putative ABC transporters were significantly up-regulated in response to Bt maize proteins (Table 6), and agree with results from a prior study for a eCry3.1Ab resistant strain of this species [75]. These contrast with other studies of *D. v. virgifera* where ABC transporter expression was not significantly different following exposures of susceptible larvae to Cry3Bb1 or Gpp34/Tpp35Ab1 pesticidal proteins [76], or where transcripts were not detectable in gut tissues [74]. Differences in ABC transporter transcription may be dependent upon environmental conditions or genetic background of strains being compared, although using an inbred strain may have minimized effects of the latter in our study.

Evidence from other systems indicate that ABC transporters are involved in pro-survival stress response mechanisms among mammals [90, 91]. Specifically, ABCC subfamily members function with glutathione S-transferases (GSTs) and UDP-galactosyl transferases (UGTs) to enhance drug and carcinogen efflux in cellular maintenance of homeostasis in human and mouse [92–94]. Our predicted up-regulation of transcripts encoding GST, UGT, and ABCC transporter domains in *D. v. virgifera* following Cry3Bb1 and Gpp34/Tpp35Ab1 exposure (Table 5), and significant enrichment for GO MF category drug metabolism and BP category drug metabolism (Fig. 3; Fig. 5), could suggest increased cellular transport may be involved in responses to Bt intoxication. ABC transporters also have other cellular functions within insects including immune responses [95], consistent with up-regulation of unique ABCC members in responses to entomopathogens (Table 6). Although mutations in a specific ABC transporter may inhibit pesticidal protein pore formation in resistant insects, proteins in the same superfamily may mediate other cellular responses following pore formation in susceptible insects. It may be possible that modulation of ABC transporter expression impacts cellular efflux capacities in response to increased solute influx through pores, but this hypothesis requires additional investigation.

### Modulation of metabolic and detoxification pathways

Our analyses showed that the most significantly enriched GO terms among differentially expressed transcripts following exposure of *D. v. virgifera* to Cry3Bb1 maize encompassed extracellular space and microbody (category CC), binding and transport functions (MF), and small molecule catabolism and drug metabolism (BP) (Fig. 3a). Transcripts significantly differentially-expressed in the Gpp34/Tpp35Ab1 maize treatment were most enriched for terms microbody, ER membrane, and extracellular space (category CC), coenzyme binding (MF), and small molecule catabolism (BP) (Fig. 3b). In conjunction with prior studies [75, 76], our results suggest an increase in metabolic processes may be a common response among *D. v. virgifera* to Bt intoxication. In other organisms, metabolic rates increase during instances of cellular repair and survival [96, 97], which might be conserved and potentially explanatory of the observed increase in metabolic pathway gene expression in susceptible *D. v. virgifera* responses to Cry protein intoxication. Also, enrichment of GO terms for extracellular space shared between Cry3Bb1 and Gpp34/Tpp35Ab1 treatments here and in eCry3.1Ab resistant *D. v. virgifera* [75], might suggest a role of secreted factors in intoxication responses.

Even though no GO enrichment analyses were conducted in a prior investigation of Cry3Bb1 maize exposure among susceptible *D. v. virgifera* larvae [74], these authors described significant differential expression of transcripts putatively encoding proteins involved in xenobiotic stress responses and detoxification (cytochrome P450 monooxygenase, esterase, oxidase, and peroxidase) functions, and transporter activities. Our study similarly predicted PFAM domains for sugar transporter and detoxification enzymes (cytochrome P450, carboxyesterase, glutathione S transferase, and UDP glycosyltransferases) are prevalent among transcripts differentially regulated in Cry3Bb1 exposed larvae (Table 5). Our data also show that sugar transporter and cytochrome P450 domains encoded by transcripts differentially-regulated in the Gpp34/Tpp35Ab1 treatment are shared between the Cry3Bb1 treatment. Coincidence of these functions between this prior study [74] and our current study suggest a role for these proteins in cellular intoxication response. Cytochrome P450s are involved in a large breadth of insect cellular functions [98], including regulation of insect ecdysone and juvenile hormone pathways [99], cuticle formation [100], and xenobiotics detoxification [101]. Uncoupling of P450 oxygenation reactions results in production of reactive oxygen species (ROS), hydrogen peroxide and superoxide [102]. Cellular homeostasis can become disrupted during times of cell stress when excess ROS is produced due to high P450 activity. ROS can trigger apoptosis [103], or act as second messengers that modulate other cellular processes [104]. Stress responses triggered by ROS during high metabolic states are intimately tied to increased ABC transporter activities [105], suggesting a possible basis for our predicted up-regulation of ABC transporters in Cry protein and entomopathogen responses (Table 6). This also highlights the value of identifying transcripts putatively involved in general (i.e. not Bt-specific) cellular stress. Moreover, we hypothesize that increases in metabolic, transport, and detoxification pathways following Bt exposure of susceptible insects may be connected with the increased energy demands of cellular repair or death/survival processes. Despite the tantalizing connections, further research is required to demonstrate the roles of these pathway components for cellular or organismal survival.

### Up-regulation of cell survival pathways

The current study predicts that two *D. v. virgifera* transcripts putatively encoding orthologs of *D. melanogaster* effector caspase DECAY and initiator caspase STRICA or DAMM (Fig. 6**)** are up-regulated following feeding on Cry3Bb1 and Gpp34/Tpp35Ab1 maize roots (Table 7). Although DRONC and effectors DRICE and DCP1 are the main caspases involved in apoptosis, DECAY and DAMM may represent redundancies or have yet unknown functions [106]. Regardless, the up-regulation of caspase-encoding transcripts in *D. v. virgifera* following Bt intoxication could suggest an apoptotic response. This is in partial agreement with prior studies showing significant up-regulation of transcripts encoding caspases in *Manduca sexta* cells following Cry1Ac exposure [67] and in midgut tissues of *S. exigua* following a sublethal exposure to the Bt Vip3A protein [68].

Because caspase activation by the apoptosome is a critical step in determining cell fate, this process is tightly regulated. In mammals, caspase translation occurs as inactive pro-peptides that undergo autocatalysis in response to pro-apoptotic stimuli [107]. However, evidence suggests *D. melanogaster* caspases are translated in active forms but are suppressed following reversible binding by inhibitor of apoptosis protein (IAP) family members [108]. Although several mechanisms function to regulate pro-apoptotic signals, only the evolutionarily conserved IAPs inhibit caspase function through direct binding [108, 109]. Paralogs IAP1 or IAP2 are each sufficient to inhibit cell death in lepidopteran cells [110], suggesting their functional conservation. IAPs are up-regulated in response to cell stress conditions [111], as was observed here following exposures of susceptible *D. v. virgifera* to Cry3Bb1 and Gpp34/Tpp35Ab1 (Table 7). Interestingly, the up-regulation of caspase- and IAP-encoding transcripts was concurrent, perhaps suggesting an intimate balance between pro- and anti-apoptotic signals within cells following Bt intoxication. For example, caspase-mediated apoptosis could be partially suppressed through the up-regulation of IAPs. Although *Drosophila* IAP1 transcription is regulated by factors including those in the Hippo pathway [112], the conservation of this regulatory framework across arthropods remains unknown. Future investigation of the basis and consequences of IAP up-regulation in *D. v. virgifera* may clarify the role of apoptosis in determining cell fate, and organismal survival following low-dose Cry intoxication.

Transcripts encoding anti-apoptotic proteins including structurally and functionally conserved transmembrane B-cell-lymphoma protein 2 (Bcl-2)-associated X (BAX) inhibitor 1 motif (TMBIM)-containing protein family members, the BAX inhibitor 1 (BI-1) and Lifeguard 4 (LFG4) [113], are significantly up-regulated in Cry3Bb1 and Gpp34/Tpp35Ab1 exposed susceptible *D. v. virgifera* larvae (Table 7). Similarly, the serine protease inhibitor, stress-associated ER protein 2 (SERP2), is also up-regulated. These factors function in cellular adaptation to stress in the mitochondrion, Golgi or endoplasmic reticulum (ER), thereby suppressing apoptosis. Specifically, Bcl-2 protein family members are critical for regulation of the intrinsic pathway of apoptosis in mammals [114]. In this system, pro-apoptotic Bcl-2 proteins, including the BAX protein, can oligomerize under cell stress conditions to form mitochondrial outer membrane pores that release cytochrome c and other factors, which in turn cause caspase activation. In *Drosophila*, there is a single pro-apoptotic Bcl-2 protein, *decbl,* that is a functional BAX ortholog [115], but evidence suggests it has a limited role in triggering apoptosis [116]. TMBIM proteins modulate stress through several intracellular mechanisms [117]. For example, BI-1 does not inhibit BAX via direct protein-protein interaction [118, 119], but instead inhibits ROS production in the mitochondrion. In the ER, BI-1 remediates the unfolded protein response (UPR) and H+ antiporter activity, counteracting cytosolic acidification characteristic of apoptosis [120]. This may be important because prolonged presence or high accumulation of misfolded proteins can lead to pro-apoptotic signaling, and the UPR can restore ER homeostasis [121]. Therefore, BI-1 promotes cell survival pathways by suppressing factors that would otherwise promote apoptotic signaling [122]. SERP also functions within the UPR by enhancing protein stability [123, 124]. Somewhat analogously, LFG4 remediates stress in the Golgi and ER [117], where the single ortholog in *D. melanogaster* interacts with the anti-apoptotic Bcl-2 protein, *buffy,* resulting in organismal survival via repression of pro-apoptotic *decbl* [125]. BI-1 and LFG4 structure and function are remarkably conserved, where ectopic expression of viral orthologs can rescue knockdown phenotypes in mammalian cells [113], suggesting retention of ortholog function in *D. v. virgifera*. Although the up-regulation of transcripts encoding TMBIM members BI-1 and LFG4, as well as SERP4, in susceptible *D. v. virgifera* following Cry3Bb1 and Gpp34/Tpp35Ab1 maize exposure (Table 7) suggests activation of pathways to counteract cell death by apoptosis or related mechanisms, additional investigations are required to determine individual contributions and impacts on cellular and organismal outcomes.

## Conclusions

A greater understanding of cellular responses among susceptible insects to pesticidal protein exposure can inform future research into mechanisms that lead to resistance. To date mutations in cell surface receptors have mainly been implicated in pesticidal protein resistance among insects, although some evidence suggests alteration of intracellular signaling or cell recovery mechanisms may have a role (see Introduction). Only a few prior studies demonstrated apoptotic pathways in Bt protein response among insects, specifically involving up-regulation of caspases [67, 68] or the MAPK p38 pathway [69, 70]. We provide evidence for an apoptotic response in susceptible *D. v. virgifera* larvae following exposure to transgenic maize roots expressing either Bt Cry3Bb1 or Gpp34/Tpp35Ab1. Interestingly, the patterns we observed in differential expression suggest counteracting pathways may simultaneously remediate cell stress and suppress apoptosis, possibly through modulating a balance between opposing pathways to determine cell fate. Because we used whole larvae, we cannot predict any possible tissue- or cell type-specific responses. In general, exposure level has a role in cell response to pore forming proteins suggesting a “high-dose” leads to death by oncosis (cell swelling and blebbing due to osmotic imbalance) as opposed to a “low-dose” that tends to trigger apoptosis [126]. Therefore, the responses of *D. v. virgifera* to the “low-dose” pesticidal protein exposures characteristic of current transgenic Bt maize hybrids commercialized for their control may not be comparable to responses among species of Lepidoptera that feed on crop tissues that provide a “high-dose”. Regardless, this work provides a framework for understanding cellular responses to Bt pesticidal protein exposure in the most devastating maize pest in the United States, and suggests that mechanisms promoting and counteracting apoptosis may characterize these responses.

## Methods

### Samples, treatments, and collections

A Bt susceptible non-diapausing strain of *D. v. virgifera* [127] maintained at the United States Department of Agriculture, Agricultural Research Service, North Central Agricultural Research Laboratory (USDA-ARS, NCARL), Brookings, SD, USA was used to generate an inbred line, Ped12. Ped12 was initiated from a single mated pair, followed by inbreeding for 9 generations: single pair full-sib mating in the F_1_ to F_5_; (generations G1 to G5), followed by *en masse* mating among siblings within G6 and G7, then single pair full-sib mating in the subsequent generations (G7 to G9). Ped12 was maintained thereafter as a closed colony of approximately 1000 individuals for 8 generations prior to use in this study.

Ped12 individuals were sampled at different developmental stages or following different exposure conditions. Different developmental stages (conditions C1 through C12; Table 1) were sampled among Ped12 individuals reared using standard laboratory methods [128] at USDA-NCARL. For conditions C13 to C16 (Table 1) maize seedling mats were germinated from seeds of hybrids expressing modified mCry3A (Event MIR604; Syngenta, Basel, Switzerland;), Cry3Bb1 (Event Mon88017 by Bayer Crop Sciences/Monsanto Company, St. Louis, MO, USA in VT TriplePro, VT3 hybrid DCK61–69 from DeKalb Seed, DeKalb, IL, USA), Gpp34/Tpp35Ab1 (Event DAS-59122-7 by Corteva/Pioneer, Indianapolis, IN, USA in HerculexXtra hybrid 2 T789 from Mycogen Seeds, San Diego, CA, USA), or no Bt (hybrid 38B85, Corteva/Pioneer). In brief, recently laid Ped12 *D. v. virgifera* eggs were suspended in a 0.15% agar solution and dispensed into 15 by 10-cm plastic containers (708 ml; The Glad Products Company, Oakland, CA) at a rate of 500 eggs per container. Containers with eggs were filled with 20 ml of water and 150 ml of the soil mixture described previously. After 1 wk., 50 maize seeds were added to containers and covered with an additional 300 ml of soil mixture and 80 ml of water. Containers were held in a controlled environmental chamber (Powers Scientific Inc., Pipersville, PA) at constant 25 °C and a photoperiod of 14:10 (L:D) h, as described previously [129]. Four weeks after infestation of eggs, larvae of each treatment were recovered from seedling mats, placed in tin foil packages, flash frozen in liquid nitrogen, and stored at − 80 °C. Seedling mats in treatments C13 to C15 received regular watering. Condition C16 simulating drought stress of hybrid 38B85 **(**Corteva/Pioneer) received no water (Table 1).

Additionally, Ped12 larvae were separately exposed to the entomopathogenic nematode *Heterorhabditis bacteriophora* (Hb) strain BU (Becker Underwood, Ames, IA, USA; C17 in Table 1), and the entomopathogenic fungus, *Metarhizium anisopliae* (Ma) strain F52 (provided by Stefan Jaronski, USDA-ARS; C18 in Table 1). In brief, 300 μl of a 510 Hb ml^− 1^ suspension was aliquoted onto filter paper in 10 cm Petri plates, and six 2nd and 3rd instars were added and exposed for 48 h. Analogously, 300 μl of 1.5 × 10^7^ Ma conidia ml^− 1^ in 0.1% Tween 20 was aliquoted onto filter paper in 10 cm Petri plates, and six 2nd and 3rd instars exposed for 48 h (C18). Adults were exposed in a modified glass scintillation vial exposure assay [130]. For this, 20 ml vials were coated with sublethal levels of the neonicotinoid insecticide, thiamethoxam (Poncho, Bayer Crop Sciences, Leverkusen, Germany; C19 in Table 1) or the adult attractant cucurbitacin (Sigma-Aldrich, St. Louis, MO, USA; C20 in Table 1). Ten Ped12 adults were exposed per vial for 24 h. In all cases, individuals were pooled as a single replicate per condition, flash frozen in liquid nitrogen, and stored at − 80 °C.

Ped12 individuals were also subjected to different treatments in triplicate and sampled for quantitative analyses of transcript expression (Table 2) and inclusion within the reference transcriptome assembly (Fig. 1). Pooled samples were collected from Ped12 eggs in diapause (treatment 1; T1), and 1st (T2) and 3rd instars (T7; Table 2) reared under standard laboratory conditions [128] at USDA-NCARL. Second instar Ped12 were exposed to maize seedling roots expressing Gpp34/Tpp35Ab1 (Herculex® XTRA Corteva/Pioneer Event DAS-59122-7 in hybrid 2 T789 from Mycogen Seed, San Diego, CA, USA; T5), Cry3Bb1 (VT TriplePro; Bayer Crop Sciences/Monsanto Company; T8), or the non-Bt hybrid 38B85 (Corteva/Pioneer; T7) as described above. Treatment 4 (T4; Table 2) consisted of larval midgut tissue dissected and pooled from approximately 50 3rd instars feeding on the non-Bt hybrid 38B85. Treatments 3 (T3) and 6 (T6) were from Ped12 larvae exposed to *M. anisopliae* and *H. bacteriophora* respectively, as described above. All pooled samples were flash frozen in liquid nitrogen separately within replicates for each treatment, and stored at − 80 °C.

### Complementary DNA libraries, sequencing, and data processing

Total RNA was purified by replicate for each condition (C1 to C20 in Table 1), and triplicates within each treatment (T1 to T8 in Table 2). Each sample was ground in liquid nitrogen and then 10.0 mg of tissue added to 250 μl TriZol Reagent (Life Technologies, Grand Island, NY, USA). Purification was conducted using the Direct-zol RNA MiniPrep kit (Zymo Research, Irvine, CA, USA), which included a 15 min DNase I digestion performed according to manufacturer instructions. Total RNA extracts were quantified using Qubit dsDNA HS Assay Kits (Life Technologies) on a Qubit 2.0 Fluorimeter (Thermo-Fisher, Wilmington, DE), and quality determined by electrophoresis on a 10 cm 2% denaturing agarose gel in 1X MOPS buffer run at 70 V for 45 min.

Normalized cDNA was prepared from an equimolar pool of total RNA isolated across 20 different conditions (C1 to C20; Table 1) using the Trimmer-2 cDNA Normalization Kit according to manufacturer instructions (Evrogen, Moscow, Russia). Resulting cDNA was quantified on a Nanodrop2000 (Thermo-Fisher). Non-normalized cDNA was prepared separately from 1.0 μg of purified RNA for each of the three replicates per treatment (T1 to T8 in Table 2) using the SMARTer cDNA Synthesis Kit (Life Technologies) according to manufacturer instructions. Long-range PCR of SMARTer cDNA products was carried out using Advantage *Taq* Polymerase according to manufacturer instructions (Life Technologies, Carlsbad, CA), which included 18 amplification cycles according to manufacturer instructions on a Tetrad2 thermocycler (BioRad, Hercules, CA, USA) with 10 min extension at 65 °C. All amplified cDNAs were quantified using dsDNA HS Assay Kits (Life Technologies) on a Qubit 2.0 Fluorometer (Thermo-Fisher).

The normalized and non-normalized cDNA samples were sent to the Laboratoire de Sequencage, Institute de Genomique, France, where uniquely indexed RNA-seq libraries were prepared using TruSeq RNA library Prep Kit V.1 (Illumina Inc., San Diego, CA, USA). Sequencing was performed on an Illumina HiSeq2000 platform (Illumina, Inc.) in 100 bp paired-end (PE) mode. Non-normalized libraries (8 conditions × 3 biological replicates per condition; *n* = 24; Table 2) were run on three different lanes of the same flow cell, with one replicate from each condition per lane. The normalized pooled library was run on a single lane of a separate Illumina HiSeq2000 flow cell. All raw reads were trimmed to remove Illumina adaptor and low-quality sequences with Phred33 bases quality scores (*q*) < 20 using Trimmomatic v 0.32 [131]. Long poly(A/T) stretches were removed using SEQCLEAN (Dana-Farber Cancer Institute, Boston, MA, USA; https://sourceforge.net/projects/seqclean/files/) with the *-l* option to retain trimmed reads ≥30 bp. Ambiguous nucleotide bases (‘N’) were removed using a custom in-house PERL script.

### De novo reference transcriptome assembly and annotation

The *D. v. virgifera* reference transcriptome assembly pipeline used an iterative approach with in silico read normalization (Fig. 1**a**). Specifically, trimmed reads from the pooled normalized library (*n* = 1) and each replicate of all non-normalized RNA-seq libraries (*n* = 24) were combined into PE and single end (SE) read groups. In silico normalization was conducted separately for each using the script *insilico_read_normalization.pl* (available in TRINITY v2014-07-17 software package [132] with parameter *--max_cov 30*. This normalization step was performed to reduce the coverage of highly expressed transcripts, thereby improving the ability to assemble transcripts expressed at low levels. In silico normalized trimmed PE and SE reads were then merged across all libraries into a single fastq file, and used in a multiple k-mer assembly approach with VELVET 1.2.03 [133] and OASES 0.2.06 [134]. K-mer hashes were prepared for k = 61, 71, 81 and 91 with in silico normalized SE and PE reads applied as *Short* and *Short Paired fastq* input classes, respectively. After each single k-mer assembly, CD-HIT-EST [135] was used to remove shorter redundant transcripts when entirely covered by other transcripts with > 99% identity. Transcripts ≥ 100 bp from the four k-mer runs were then merged into a combined assembly using *kmerge*. CD-HIT-EST was once again used to remove the shorter redundant transcripts (same parameters as previously). To remove additional residual redundancy, we used a custom tool which merges two sequences using successive iterations of CAP3 [136] and NRCL tools with a progressive reduction in the stringency parameters [137]. The final threshold included shared ≥ 94% identity with ≥ 100 bp overlap and overhangs < 40 bp. The final set of transcripts were filtered to remove those < 200 bp to satisfy NCBI Transcriptome Shotgun Assembly (TSA) minimum requirements.

Completeness of the de novo *D. v. virgifera* reference transcriptome was evaluated by ortholog identification using the Core Eukaryotic Genes Mapping Approach (CEGMA) [138]. Transcripts were mapped to 248 CEGs, and the set of 1066 universal single copy orthologs from Arthropoda obtained from OrthoDB v 9 [139] as identified using BUSCO v 3 [140] (*E*-value cutoff ≤ 0.05).

Assembled transcripts were annotated by comparing to the SWISSPROT database, and to predicted protein databases from gene models for insects (*Tribolium castaneum* v3.0, *Dendroctonus ponderosae*, *A. glabripennis*, *Drosophila melanogaster* r5.46), nematode (*H. bacteriophora*), fungus (*M. anisopliae*), and NCBI *Wolbachia* protein sequences. In all cases the BLASTx algorithm [141] was used for querying, and the results filtered for those with an *E*-value ≤ 10^− 7^ and high-scoring segment pairs (HSP) length ≥ 25 amino acids. Putative transcript origin of *H. bacteriophora*, *M. anisopliae*, or *Wolbachia* were predicted according to the lowest *E*-value obtained from the set of database query results. Transcripts were considered “full-length” if query match lengths covered the entire sequence of the best-hit protein. Twenty missing amino acids were allowed in HSPs at both ends of the subject if the query length was compatible with the complete the sequence. Among the remaining *D. v. virgifera* transcripts, a “near complete” bin was defined as those with a best HSP that covered ≥ 80% of the subject length. Putative peptide-coding region (CDS) and derived protein sequences were predicted among all assembled *D. v. virgifera* transcripts using FRAMEDP [142]. Putative protein family domains were assigned by searches of the PFAM A database v. 27.0 [143, 144] using the program HMMSEARCH [145] with derived *D. v. virgifera* proteins as the queries.

### Estimates of quantitative differences in transcript expression

Transcript abundances (gene expression levels) were estimated by comparing non-normalized read counts among treatments (T1 to T8; Table 2), which relied on an experimental design and analysis pipeline that accounted for variance among replicates within and between treatment (Fig. 1**b**). To accomplish this, trimmed non-normalized read data from 3 replicates within treatment (T1 to T8; Table 2; *n* = 24) were mapped separately to the de novo assembled *D. v. virgifera* reference transcriptome (above) using the BOWTIE2 aligner v2.1.0 [146] with parameters *--all* (report all alignments), *--end-to-end* (entire read must align), and *--sensible* (0 mismatches allowed in a seed of 22 bp). Mapped reads were filtered using “high stringency” parameters (PE and SE reads were retained if mapped properly on only one transcript. In the case of PE reads, both left and right reads were required to map in opposite directions on the same transcript at a distance compatible with the expected mean size of the fragment). The resulting output files were parsed by an in-house script to count the number of reads that mapped to each transcript.

Significant differences in aligned read counts (gene expression) were predicted for the comparisons of treatment T7 (Cn maize) with treatments T3 (Ma), T6 (Hb), T5 (Hx) or T8 (VT3) using the R packages DESeq2 v1.6.3 [147] and EdgeR v3.8.6 [148]. Other possible comparisons were not conducted. DESeq2 fit the dispersion of read counts for each transcript to an empirical mean, and performed independent filtering (default alpha = 0.1) to remove transcripts with low read counts (baseMean), which are subject to greater uncertainty and influence the results of multiple testing. *P*-values were adjusted in both packages for a false discovery rate (FDR) among multiple comparisons using the Benjamini-Hochberg (BH) method [149]. For each comparison with an FDR ≤ 0.05 were considered significant for DESeq2 and EdgeR, and the final set of differentially expressed genes was defined as those with an FDR ≤ 0.05 using both statistical methods.

### Differential expression following Cry3Bb1 and Gpp34/Tpp35Ab1exposure

Linux *awk* and *uniq* commands were used to compile a dataset of transcripts differentially expressed in both Cry3Bb1 and Gpp34/Tpp35Ab1 treatments compared to controls (Cn). The same methods were used to identify and filter transcripts that were differentially expressed in entomopathogen Hb and/or Ma treatments compared to controls, and that were also shared with those differentially expressed in one or more of the treatments 1) Cry3Bb1 vs Cn 2) Gpp34/Tpp35Ab1 vs Cn, and 3) both Cry3Bb1 and Gpp34/Tpp35Ab1 vs Cn. This filtering excluded transcripts not specific to Bt toxin Cry responses.

Putative cellular component (CC), molecular function (MF), and biological process (BP) categories were assigned to differentially expressed transcripts using the PFAM domains to retrieve corresponding gene ontologies (GOs). This was accomplished using the dcGOR 1.0.6 package [150]. Subsequent enrichment analyses by dcGOR 1.0.6 at GO level 2 applied significance thresholds of *E*-values ≤ 1.0^− 5^ for CC and MF terms, and ≤ 1.0^− 6^ for BP. Corresponding *E*-values and total number of transcripts represented within each GO category at level 2 were plotted for the comparisons 1) Cry3Bb1 vs Cn, 2) Gpp34/Tpp35Ab1 vs Cn, and 3) both Cry3Bb1 and Gpp34/Tpp35Ab1 vs Cn. Differentially expressed transcripts with ABC transporter PFAM domains were assigned putative orthologs as described previously [151]. Putative tetraspanin-like proteins translated from differentially expressed transcripts DIAVI004770 and DIAVI021979 were used as BLASTp queries to the NCBI nr protein database and flybase [152]. This was repeated for *H. armigera* TSPAN1.

### Phylogenetics and structural annotations

Protein sequences of *Drosophila melanogaster* caspases were downloaded from FlyBase [152] by gene name search: DRONC, DRED, DAMM, STRICA, DECAY, DCP-1, and DRICE, [153]. The 28,061 RefSeq proteins derived from gene models in the annotated draft *D. v. virgifera* genome assembly GCA_003013835.2 Dvir_v2 (NCBI GenBank Accession PXJM00000000.2) were downloaded in fasta format, and loaded into a local BLAST database. This database was queried with protein sequences for *D. melanogaster* caspases using the BLASTp algorithm [141] (*E*-value cutoffs ≤ 10^− 20^). An analogous search was conducted against the 56,656 proteins predicted from all assembled transcripts. A multiple protein sequence alignment was generated among *D. melanogaster* and *D. v. virgifera* caspase catalytic domains using the Clustal W algorithm [154] within the MEGA8.0 alignment utility [155] (default parameters). The LG + G + I model of protein sequence evolution [77] maximized the BIC score when alignment gaps were ignored, and was subsequently used to construct a ML-based phylogeny with a consensus built from among 1000 bootstrap pseudo-replicates using MEGA8.0 [155].

Sequences from up-regulated transcripts encoding putative inhibitor of apoptosis proteins (IAPs; DIAVI011972 and DIAVI007715), B-cell-lymphoma protein 2 (Bcl-2)-associated X (BAX) inhibitor (BI) proteins BI-1 (DIAVI026079) and Lifeguard 4-like (LFG4; DIAVI029891), and stress-induced endoplasmic reticulum protein 2 (SERP2) were used to query the local database of 28,061 Dvir_v2 RefSeq proteins using the BLASTp algorithm [140] (*E*-value cutoffs ≤ 10^− 60^). In addition to PFAM and GO annotations (above), further structural annotation and position of functional domains and residues were predicted by query of the conserved domain database (CDD) [156] using default parameters. Multiple protein sequence alignments were also generated for *D. v. virgifera* IAP, and BI-1 and LFG4 proteins with orthologs from *D. melanogaster*, and other coleopteran species (*Tribolium castaneum, Dendroctonus ponderosae, A. glabripennis* and *Leptinotarsa decemlineata*) using the Clustal W algorithm. Classification of *D. v. virgifera* IAP protein families were based on that defined for *D. melanogaster* orthologs [109]. Prediction of transmembrane region (TMR) for *D. v. virgifera* BI-1 and LFG4 applied a Hidden Markov Model using the default parameters of the application, TMHMM 2.0 [157].

## Supplementary Information

**Additional file 1: Supplementary Table S1.**. Trimmed reads obtained from Illumina sequencing libraries.

**Additional file 2: Supplementary Fig. S1.** Putative orthology of assembled transcripts in the *Diabrotica virgifera virgifera* reference transcriptome. Results based on BLASTx hits to protein models from *Drosophila melanogaster* (Dm), *Tribolium castaneum* (Tc), *Dendroctonus ponderosae* (Dp), and *Anaplophora glabripennis* (Ag). Number of unique *D. v. virgifera* derived proteins with shared evidence across all comparisons (*n* = 12,474) and all coleopteran species (*n* = 3883) are highlighted. A total of 34,684 assembled *D. v. virgifera* transcripts had ≥ 1 hit across all sets of reference protein models.

**Additional file 3: Supplementary Table S2.** Number of trimmed Illumina single-end (SE) and paired-end (PE) reads that aligned to the *Diabrotica virgifera virgifera* reference transcriptome across replicates of each RNA-seq library. 1X = reads that aligned uniquely; > 1X = reads that aligned to greater than once location.

**Additional file 4: Supplementary Table S3.** Transcripts with significant differences in read counts (expression) between *Diabrotica virgifera virgifera* larvae feeding on transgenic maize expressing the insecticidal *B. thuringiensis* (Bt) Cry3Bb1 toxin (T8; Table 2) compared to control non-Bt maize (T7). For each transcript, indication of differential expression also being shared when larvae were exposed to *Heterorhabditis bacteriophora* (Hb) and *Metarhizium anisopliae* (Ma) is indicated with a “1”. Standard output are shown for DESeq2 (baseMean = the average of the normalized counts taken over all samples; log2FoldChange = log2 fold change between the groups; lfcSE = standard error of the log2FoldChange; stat = Wald statistic; pvalue = Wald test *P*-value; BH_padj = Benjamini-Hochberg adjusted p-value) and EdgeR (logFC = log2 fold change between the groups; logCPM = the average log2-counts-per-million; PValue = the two-sided *P*-value; BF_FDR = Bonferroni adjusted *P*-value). Lack of significance for any given transcript in DESeq2 or EdgeR results shown as “-”. The 1055 transcripts differentially expressed in both DESeq2 and EdgeR estimates are shown above the horizonal line. Transcript annotations include presence (Y) or absence (N) of signal_peptide probability (signalp_prob) threshold of > 0.700 shown only for “complete” proteins (match length = 1.0 to *Drosophila melanogaster* (Dm) or *Trobiolium castaneum* (Tc) protein models). PFAMSCAN_PfamA search results (format transcript query start, transcript query end: frame, strand (+ or -), range of hit to PFAM domain/PFAM domain name/ E-value/percent identity). Predicted protein information given (transcript start: transcript end: frame: strand (+ or -): amino acid sequence). BLASTx query results to indicated databases shown in standard output format with “/” indicating no values for queries receiving of hits. The 9 transcripts in blue italicized text are putatively derived from Hb or Ma.

**Additional file 5: Supplementary Table S4.** Transcripts with significant differences in read counts (expression) between *Diabrotica virgifera virgifera* larvae feeding on transgenic maize expressing the insecticidal *B. thuringiensis* (Bt) Gpp34/Tpp35Ab1 toxin (T5; Table 2) compared to control non-Bt maize (T7). For each transcript, indication of differential expression also being shared when larvae were exposed to *Heterorhabditis bacteriophora* (Hb) and *Metarhizium anisopliae* (MA) is indicated with a “1”. Standard output are shown for DESeq2 (baseMean = the average of the normalized counts taken over all samples; log2FoldChange = log2 fold change between the groups; lfcSE = standard error of the log2FoldChange; stat = Wald statistic; pvalue = Wald test *P*-value; BH_padj = Benjamini-Hochberg adjusted *P*-value) and EdgeR (logFC = log2 fold change between the groups; logCPM = the average log2-counts-per-million; PValue = the two-sided *P*-value; BF_FDR = Bonferroni adjusted *P*-value). Lack of significance for any given transcript in DESeq2 or EdgeR results shown as “-”. The 1374 transcripts differentially expressed in both DESeq2 and EdgeR estimates are shown above the horizonal line. Transcript annotations include presence (Y) or absence (N) of signal_peptide probability (signalp_prob) threshold of > 0.700 shown only for “complete” proteins (match length = 1.0 to *Drosophila melanogaster* (Dm) or *Trobiolium castaneum* (Tc) protein models). PFAMSCAN_PfamA search results (format transcript query start, transcript query end: frame, strand (+ or -), range of hit to PFAM domain/PFAM domain name/ E-value/percent identity). Predicted protein information given (transcript start: transcript end: frame: strand (+ or -): amino acid sequence). BLASTx query results to indicated databases shown in standard output format with “/” indicating no values for queries receiving of hits. The 13 transcripts in blue italicized text are putatively derived from Hb or Ma.

**Additional file 6: Supplementary Table S5.** Transcripts with significant differences in read counts (expression) between *Diabrotica virgifera virgifera* larvae infected with Heterorhabditis bacteriophora (Hb) (T6; Table 2) compared to control non-Bt maize (T7). For each transcript, standard output are shown for DESeq2 (baseMean = the average of the normalized counts taken over all samples; log2FoldChange = log2 fold change between the groups; lfcSE = standard error of the log2FoldChange; stat = Wald statistic; pvalue = Wald test *P*-value; BH_padj = Benjamini-Hochberg adjusted *P*-value) and EdgeR (logFC = log2 fold change between the groups; logCPM = the average log2-counts-per-million; PValue = the two-sided *P*-value; BF_FDR = Bonferroni adjusted *P*-value). Lack of significance for any given transcript in DESeq2 or EdgeR results shown as “-”. The 1562 transcripts differentially expressed in both DESeq2 and EdgeR estimates are shown above the horizonal line. Transcript annotations include presence (Y) or absence (N) of signal_peptide probability (signalp_prob) threshold of > 0.700 shown only for “complete” proteins (match length = 1.0 to *Drosophila melanogaster* (Dm) or *Trobiolium castaneum* (Tc) protein models). PFAMSCAN_PfamA search results (format transcript query start, transcript query end: frame, strand (+ or -), range of hit to PFAM domain/PFAM domain name/ E-value/percent identity). Predicted protein information given (transcript start: transcript end: frame: strand (+ or -): amino acid sequence). BLASTx query results to indicated databases shown in standard output format with “/” indicating no values for queries receiving of hits.

**Additional file 7: Supplementary Table S6.** Transcripts with significant differences in read counts (expression) between *Diabrotica virgifera virgifera* larvae infected with *Metarhizium anisopliae* (Ma) (T3; Table 2) compared to control non-Bt maize (T7). For each transcript, standard output are shown for DESeq2 (baseMean = the average of the normalized counts taken over all samples; log2FoldChange = log2 fold change between the groups; lfcSE = standard error of the log2FoldChange; stat = Wald statistic; pvalue = Wald test *P*-value; BH_padj = Benjamini-Hochberg adjusted *P*-value) and EdgeR (logFC = log2 fold change between the groups; logCPM = the average log2-counts-per-million; PValue = the two-sided *P*-value; BF_FDR = Bonferroni adjusted *P*-value). Lack of significance for any given transcript in DESeq2 or EdgeR results shown as “-”. The 1199 transcripts differentially expressed in both DESeq2 and EdgeR estimates are shown above the horizonal line. Transcript annotations include presence (Y) or absence (N) of signal_peptide probability (signalp_prob) threshold of > 0.700 shown only for “complete” proteins (match length = 1.0 to *Drosophila melanogaster* (Dm) or *Trobiolium castaneum* (Tc) protein models). PFAMSCAN_PfamA search results (format transcript query start, transcript query end: frame, strand (+ or -), range of hit to PFAM domain/PFAM domain name/ E-value/percent identity). Predicted protein information given (transcript start: transcript end: frame: strand (+ or -): amino acid sequence). BLASTx query results to indicated databases shown in standard output format with “/” indicating no values for queries receiving of hits.

**Additional file 8: Supplementary Fig. S2.** Dispersion of raw and DESeq2 adjusted read counts about empirical mean for comparisons of triplicate RNA-seq data within **A)** control maize (Cn; Treatment T7) and Cry3Bb1 maize (VT3; T8), and **C)** control maize (Cn; T7) and Gpp34/Tpp35Ab1 maize (Hx; T5). MA-plots of Log_2_ transformed fold-change estimates and normalized mean read count are shown for **B)** control maize (Cn; Treatment T7) and Cry3Bb1 maize (VT3; T8), and **D)** control maize (Cn; T7) and Gpp34/Tpp35Ab1 maize (Hx; T5), with datapoints (change in transcript read counts) surpassing a Benjamini and Hochberg (1995) adjusted false discovery rate (FDR) of ≤0.05 shown in red.

**Additional file 9: Supplementary Table S7.** Predicted functional PFAM domains encoded by transcripts differentially expressed by *Diabrioca virgifera virgifera* exposed to *Heterorhabditis bacteriophora* compared to controls. Counts provided for instances when ≥3 transcripts received an annotation within at least one of the treatments. InterPro identification (InterPro_ID) are also given, with NA indicating corresponding InterPro_ID not available.

**Additional file 10: Supplementary Table S8.** Predicted functional PFAM domains encoded by transcripts differentially expressed by *Diabrioca virgifera virgifera* exposed to *Metarhizium anisopliae* compared to controls. Counts provided for instances when ≥3 transcripts received an annotation within at least one of the treatments. InterPro identification (InterPro_ID) are also given, with NA indicating corresponding InterPro_ID not available.

**Additional file 11: Supplementary Fig. S3.** Gene Ontology (GO) terms enriched among transcripts differentially expressed in *Heterorhabditis bacteriophora* (Hb) and *Metarhizium anisopliae* (Ma) treatments that were shared with treatments **A)** Cry3Bb1 and **B)** Gpp34/Tpp35Ab1. Significantly overrepresented GO terms are shown within categories biological process (BP) and cellular component (CC) (FDR ≤ 1.0E^− 5^) and molecular function (MF) at level 2 (FDR ≤ 1.0E^− 7^; grey bars). Categories listed by GO ID and GO term. Number of transcripts encoding each PFAM domain within each functional category are indicated (black bars).

**Additional file 12: Supplementary Table S9.** Transcripts with significant differences in read counts (expression) between *Diabrotica virgifera virgifera* larvae feeding on transgenic maize expressing the insecticidal *B. thuringiensis* (Bt) Cry3Bb1 toxin (T8; Table 2) and Gpp34/Tpp35Ab1 (T5) compared to control non-Bt maize (T7). For each transcript, indication of differential expression also predicted when larvae were exposed to *Heterorhabditis bacteriophora* (Hb) and *Metarhizium anisopliae* (Ma) is indicated with a “1”. Standard output are shown for DeSeq2 (baseMean = the average of the normalized counts taken over all samples; log2FC = log2 fold change between the groups; lfcSE = standard error of the log2FoldChange; stat = Wald statistic; pvalue = Wald test *P*-value; BH_padj = Benjamini-Hochberg adjusted *P*-value) and EdgeR (logFC = log2 fold change between the groups; logCPM = the average log2-counts-per-million; PValue = the two-sided *P*-value; BF_FDR = Bonferroni adjusted *P*-value). Lack of significance for any given transcript in DESeq2 or EdgeR results shown as “-”. Mean log2FC among DESeq2 and EdgeR estimates are provided for each transcript. Transcript annotations include presence (Y) or absence (N) of signal_peptide probability (signalp_prob) threshold of > 0.700 shown only for “complete” proteins (match length = 1.0 to *Drosophila melanogaster* (Dm) or *Trobiolium castaneum* (Tc) protein models). PFAMSCAN_PfamA search results (format transcript query start, transcript query end: frame, strand (+ or -), range of hit to PFAM domain/PFAM domain name/ E-value/percent identity). Predicted protein information given (transcript start: transcript end: frame: strand (+ or -): amino acid sequence). BLASTx query results to indicated databases shown in standard output format with “/” indicating no values for queries receiving of hits.

**Additional file 13: Supplementary Fig. S4.** Alignment of partial enzymatic domain sequences from *Drosophila melanogaster* caspases DRONC, DRED, DAMM, STRICA, DCP-1, and DRICE (accessions in footnotes) with putative *Diabrotica virgifera virgifera* orthologs from the reference transcriptome assembly (DIAVI02NNNN) in this study and RefSeq gene models (XP_0281NNNNN) from the *D. v. virgifera* genome assembly Dvir_v2.0 (GCA_003013835.2; GenBank accession PXJM00000000.2). Conserved residues are highlighted grey, with those involved in binding and catalysis in black and yellow, respectively.

**Additional file 14: Supplementary Fig. S5.** Structural annotation of inhibitor of apoptosis proteins (IAPs) encoded by transcripts differentially expressed following *Diabrotica virgifera virgifera* exposure to Cry3Bb1 or Gpp34/Tpp35Ab1. Annotations based on structure defined by Hay et al. (2000).

**Additional file 15: Supplementary Fig. S6.** Multiple protein sequence alignment for B-cell-lymphoma protein 2 (Bcl-2)-associated X (BAX) inhibitor (BI) proteins (BI) and Lifeguard 4-like (LFG4) orthologs.

**Additional file 16: Supplementary Fig. S7.** Orthology of stress-induced endoplasmic reticulum protein 2 (SERP2) encoded by the differentially expressed transcript DIAVI057195 in *Diabrotica virgifera virgifera* larvae exposed to Cry3Bb1 and Gpp34/Tpp35Ab1.

## Data Availability

The datasets used and/or analysed during the current study are available in the National Center for Biotechnology Information (NCBI) under BioProject PRJEB28633 (https://www.ncbi.nlm.nih.gov/bioproject/?term=PRJEB28633) containing Sequence Read Archive (SRA) runs ERX2800462 to ERX2800486 (https://www.ncbi.nlm.nih.gov/sra?linkname=bioproject_sra_all&from_uid=491890). Assembled transcripts are available from the NCBI Transcriptome Shotgun Assembly (TSA; accession ERZ1775117.1). Additional data generated or analyzed in this study are included in this published article and its supplementary information files.

## Abbreviations

Bt: *B. thuringiensis*
BI-1: B-cell-lymphoma protein 2 (Bcl-2)-associated X (BAX) inhibitor-1
Cry: crystalline protein
Gpp: AeGerolysin-like pesticidal protein
LFG4: Lifeguard 4
SERP2: stress-associated ER protein 2
Tpp: toxin-10-like pesticidal protein
