# Supplementary material for: Up-regulation of apoptotic- and cell survival-related gene pathways following exposures of western corn rootworm to B. thuringiensis crystalline pesticidal proteins in transgenic maize roots
Source: BMC Genomics. 2021 Sep 4;22:639. doi: 10.1186/s12864-021-07932-4 (PMC8418000; doi:10.1186/s12864-021-07932-4)
Supplement: Supplementary file 1 — Additional file 1: Supplementary Table S1.. Trimmed reads obtained from Illumina sequencing libraries. [file 12864_2021_7932_MOESM1_ESM.docx]

**Supplementary Table S1**: Trimmed reads obtained from Illumina sequencing libraries.

| Treatment | SRA accession | Libraries | Paired end | Single end | Total |
| --- | --- | --- | --- | --- | --- |
| Pooled | ERR2791377 | Pool* | 2x 97,351,992 | 54,980,082 | 249,684,066 |
|  |  | *subtotal* | *194,703,984* | *54,980,082* | *249,684,066* |
| DiapEgg | ERR2791371 | LibA | 2x 8,219,445 | 3,879,752 | 20,318,642 |
|  | ERR2791372 | LibB | 2x 7,420,406 | 3,497,979 | 18,338,791 |
|  | ERR2791373 | LibC | 2x 9,011,721 | 3,682,443 | 21,705,885 |
|  |  | *subtotal* | *49,303,144* | *11,060,174* | *60,363,318* |
| 1st-Instar | ERR2791374 | LibD | 2x 5,817,317 | 4,886,995 | 16,521,629 |
|  | ERR2791375 | LibE | 2x 4,019,800 | 3,588,558 | 11,628,158 |
|  | ERR2791376 | LibF | 2x 12,926,197 | 7,153,534 | 33,005,928 |
|  |  | *subtotal* | *45,526,628* | *15,629,087* | *61,155,715* |
| gut | ERR2791381 | LibG | 2x 14,932,376 | 6,102,962 | 35,967,714 |
|  | ERR2791382 | LibH | 2x 16,707,732 | 7,684,965 | 41,100,429 |
|  | ERR2791383 | LibI | 2x 14,064,302 | 5,763,568 | 33,892,172 |
|  |  | *subtotal* | *91,408,820* | *19,551,495* | *110,960,315* |
| ma | ERR2791378 | LibJ | 2x 8,947,640 | 6,192,157 | 24,087,437 |
|  | ERR2791379 | LibK | 2x 5,489,083 | 5,007,533 | 15,985,699 |
|  | ERR2791380 | LibL | 2x 6,515,853 | 4,449,751 | 17,481,457 |
|  |  | *subtotal* | *41,905,152* | *15,649,441* | *57,554,593* |
| Hx | ERR2791384 | LibM | 2x 9,099,157 | 3,882,253 | 22,080,567 |
|  | ERR2791385 | LibN | 2x 8,743,241 | 5,681,714 | 23,168,196 |
|  | ERR2791386 | LibO | 2x 9,808,034 | 4,534,265 | 24,150,333 |
|  |  | *subtotal* | *55,300,864* | *14,098,232* | *69,399,096* |
| Hb | ERR2791387 | LibP | 2x 2,343,998 | 3,588,174 | 8,276,170 |
|  | ERR2791388 | LibQ | 2x 5,301,532 | 4,438,629 | 15,041,693 |
|  | ERR2791389 | LibR | 2x 4,931,524 | 3,732,371 | 13,595,419 |
|  |  | *subtotal* | *25,154,108* | *11,759,174* | *36,913,282* |
| 3rd-Instar | ERR2791390 | LibS | 2x 8,809,838 | 5,665,211 | 23,284,887 |
|  | ERR2791391 | LibT | 2x 4,768,402 | 4,590,926 | 14,127,730 |
|  | ERR2791392 | LibU | 2x 5,436,894 | 3,806,618 | 14,680,406 |
|  |  | *subtotal* | *38,030,268* | *14,062,755* | *52,093,023* |
| VT3 | ERR2791393 | LibV | 2x 6,055,938 | 3,240,268 | 15,352,144 |
|  | ERR2791394 | LibW | 2x 8,634,735 | 4,912,395 | 22,181,865 |
|  | ERR2791395 | LibX | 2x 13,687,667 | 6,802,205 | 34,177,539 |
|  |  | *subtotal* | *56,756,680* | *14,954,868* | *71,711,548* |
|  |  | ***Total*** | ***299,044,824*** | ***171,745,308*** | ***769,834,956*** |

* Equimolar quantities from each of 20 conditions (C1 to C20) in Table 1.
