# Supplementary material for: Up-regulation of apoptotic- and cell survival-related gene pathways following exposures of western corn rootworm to B. thuringiensis crystalline pesticidal proteins in transgenic maize roots
Source: BMC Genomics. 2021 Sep 4;22:639. doi: 10.1186/s12864-021-07932-4 (PMC8418000; doi:10.1186/s12864-021-07932-4)

**Supplementary Figure S1:** Putative orthology of assembled transcripts in the *Diabrotica virgifera virgifera* reference transcriptome. Results based on BLASTx hits to protein models from *Drosophila melanogaster* (Dm), *Tribolium castaneum* (Tc), *Dendroctonus ponderosae* (Dp), and *Anaplophora glabripennis* (Ag). Number of unique *D. v. virgifera* derived proteins with shared evidence across all comparisons (*n* = 12,474) and all coleopteran species (*n* = 3,883) are highlighted. A total of 34,684 assembled *D. v. virgifera* transcripts had ≥ 1 hit across all sets of reference protein models.


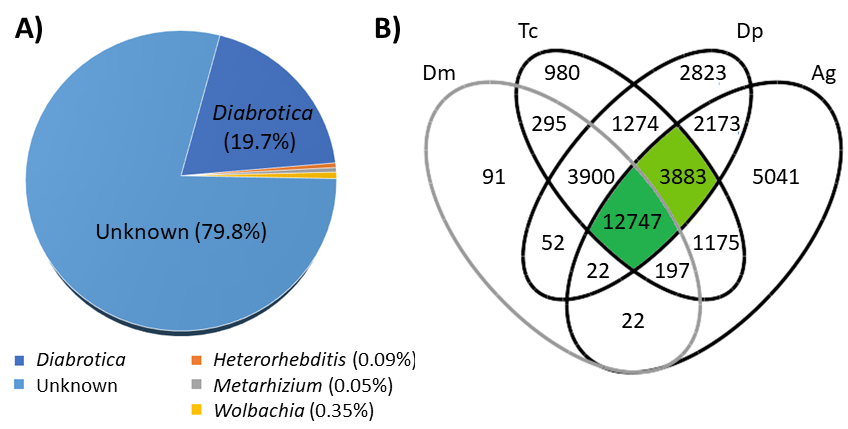

Supplement: Supplementary file 2 — Additional file 2: Supplementary Fig. S1. Putative orthology of assembled transcripts in the Diabrotica virgifera virgifera reference transcriptome. Results based on BLASTx hits to protein models from Drosophila melanogaster (Dm), Tribolium castaneum (Tc), Dendroctonus ponderosae (Dp), and Anaplophora glabripennis (Ag). Number of unique D. v. virgifera derived proteins with shared evidence across all comparisons (n = 12,474) and all coleopteran species (n = 3883) are highlighted. A total of 34,684 assembled D. v. virgifera transcripts had ≥ 1 hit across all sets of reference protein models. [file 12864_2021_7932_MOESM2_ESM.docx]
