# Supplementary material for: Up-regulation of apoptotic- and cell survival-related gene pathways following exposures of western corn rootworm to B. thuringiensis crystalline pesticidal proteins in transgenic maize roots
Source: BMC Genomics. 2021 Sep 4;22:639. doi: 10.1186/s12864-021-07932-4 (PMC8418000; doi:10.1186/s12864-021-07932-4)
Supplement: Supplementary file 3 — Additional file 3: Supplementary Table S2. Number of trimmed Illumina single-end (SE) and paired-end (PE) reads that aligned to the Diabrotica virgifera virgifera reference transcriptome across replicates of each RNA-seq library. 1X = reads that aligned uniquely; > 1X = reads that aligned to greater than once location. [file 12864_2021_7932_MOESM3_ESM.docx]

**Supplementary Table S2**: Number of trimmed Illumina single-end (SE) and paired-end (PE) reads that aligned to the *Diabrotica virgifera virgifera* reference transcriptome across replicates of each RNA-seq library. 1X = reads that aligned uniquely; >1X = reads that aligned to greater than once location.

| ID | Library | Trimmed reads | total | 1X | >1X |  | SE 1X | PE 1X | PE | Total |
| --- | --- | --- | --- | --- | --- | --- | --- | --- | --- | --- |
| T1 | LibA | 20,318,642 | 0.8880 | 0.6403 | 0.2476 |  | 0.1394 | 0.2321 | 0.1982 | 0.5697 |
|  | LibB | 18,338,791 | 0.8896 | 0.6342 | 0.2554 |  | 0.1414 | 0.2365 | 0.2127 | 0.5906 |
|  | LibC | 21,705,885 | 0.8791 | 0.6367 | 0.2423 |  | 0.1268 | 0.2315 | 0.2031 | 0.5615 |
|  |  | *subtotal 60,363,318* |  |  |  |  |  |  |  |  |
| T2 | LibD | 16,521,629 | 0.8059 | 0.5850 | 0.2208 |  | 0.1926 | 0.1768 | 0.1204 | 0.4898 |
|  | LibE | 11,628,158 | 0.8378 | 0.6078 | 0.2300 |  | 0.1957 | 0.1973 | 0.1336 | 0.5266 |
|  | LibF | 33,005,928 | 0.8649 | 0.5997 | 0.2653 |  | 0.1377 | 0.2153 | 0.1882 | 0.5412 |
|  |  | *subtotal 61,155,715* |  |  |  |  |  |  |  |  |
| T3 | LibG | 24,087,437 | 0.8579 | 0.6831 | 0.1748 |  | 0.1929 | 0.2273 | 0.0866 | 0.5068 |
|  | LibH | 15,985,699 | 0.8417 | 0.5996 | 0.2420 |  | 0.1873 | 0.1963 | 0.1294 | 0.5130 |
|  | LibI | 17,481,457 | 0.8625 | 0.6497 | 0.2128 |  | 0.1660 | 0.2207 | 0.1256 | 0.5122 |
|  |  | *subtotal 57,554,593* |  |  |  |  |  |  |  |  |
| T4 | LibJ | 35,967,714 | 0.9225 | 0.7207 | 0.2018 |  | 0.1364 | 0.279 | 0.1177 | 0.5331 |
|  | LibK | 41,100,429 | 0.9249 | 0.7268 | 0.1981 |  | 0.1537 | 0.2874 | 0.1101 | 0.5512 |
|  | LibL | 33,892,172 | 0.9219 | 0.7317 | 0.1902 |  | 0.1367 | 0.2852 | 0.1076 | 0.5295 |
|  |  | *subtotal 110,960,315* |  |  |  |  |  |  |  |  |
| T5 | LibM | 22,080,567 | 0.8853 | 0.7279 | 0.1575 |  | 0.1455 | 0.2785 | 0.0897 | 0.5138 |
|  | LibN | 23,168,196 | 0.8752 | 0.6730 | 0.2022 |  | 0.1715 | 0.2442 | 0.1164 | 0.5302 |
|  | LibO | 24,150,333 | 0.9006 | 0.7013 | 0.1993 |  | 0.1338 | 0.2703 | 0.1206 | 0.5247 |
|  |  | *subtotal 69,399,096* |  |  |  |  |  |  |  |  |
| T6 | LibP | 8,276,170 | 0.7213 | 0.5231 | 0.1983 |  | 0.2760 | 0.0801 | 0.0475 | 0.4036 |
|  | LibQ | 15,041,693 | 0.8557 | 0.6805 | 0.1752 |  | 0.2155 | 0.2235 | 0.0826 | 0.5216 |
|  | LibR | 13,595,419 | 0.8200 | 0.6139 | 0.2060 |  | 0.1735 | 0.2006 | 0.1048 | 0.4788 |
|  |  | *subtotal 36,913,282* |  |  |  |  |  |  |  |  |
| T7 | LibS | 23,284,887 | 0.8362 | 0.5716 | 0.2646 |  | 0.1421 | 0.1955 | 0.1721 | 0.5097 |
|  | LibT | 14,127,730 | 0.8249 | 0.6220 | 0.2029 |  | 0.2200 | 0.188 | 0.0976 | 0.5056 |
|  | LibU | 14,680,406 | 0.8221 | 0.5998 | 0.2224 |  | 0.1678 | 0.1947 | 0.1306 | 0.4931 |
|  |  | *subtotal 52,093,023* |  |  |  |  |  |  |  |  |
| T8 | LibV | 15,352,144 | 0.8683 | 0.6425 | 0.2258 |  | 0.1316 | 0.2422 | 0.1439 | 0.5177 |
|  | LibW | 22,181,865 | 0.8985 | 0.7506 | 0.1480 |  | 0.1920 | 0.2660 | 0.0698 | 0.5278 |
|  | LibX | 34,177,539 | 0.9079 | 0.7005 | 0.2074 |  | 0.1453 | 0.2651 | 0.1316 | 0.5420 |
|  |  | *subtotal 71,711,548* |  |  |  |  |  |  |  |  |
|  |  |  |  |  |  |  |  |  |  |  |
