# Supplementary material for: Up-regulation of apoptotic- and cell survival-related gene pathways following exposures of western corn rootworm to B. thuringiensis crystalline pesticidal proteins in transgenic maize roots
Source: BMC Genomics. 2021 Sep 4;22:639. doi: 10.1186/s12864-021-07932-4 (PMC8418000; doi:10.1186/s12864-021-07932-4)

**Supplementary Figure S2:** Dispersion of raw and DESeq2 adjusted read counts about empirical mean for comparisons of triplicate RNA-seq data within **A)** control maize (Cn; Treatment T7) and Cry3Bb1 maize (VT3; T8), and **C)** control maize (Cn; T7) and Gpp34/Tpp35Ab1 maize (Hx; T5). MA-plots of Log_2_ transformed fold-change estimates and normalized mean read count are shown for **B)** control maize (Cn; Treatment T7) and Cry3Bb1 maize (VT3; T8), and **D)** control maize (Cn; T7) and Gpp34/Tpp35Ab1 maize (Hx; T5), with datapoints (change in transcript read counts) surpassing a Benjamini and Hochberg (1995) adjusted false discovery rate (FDR) of ≤ 0.05 shown in red.


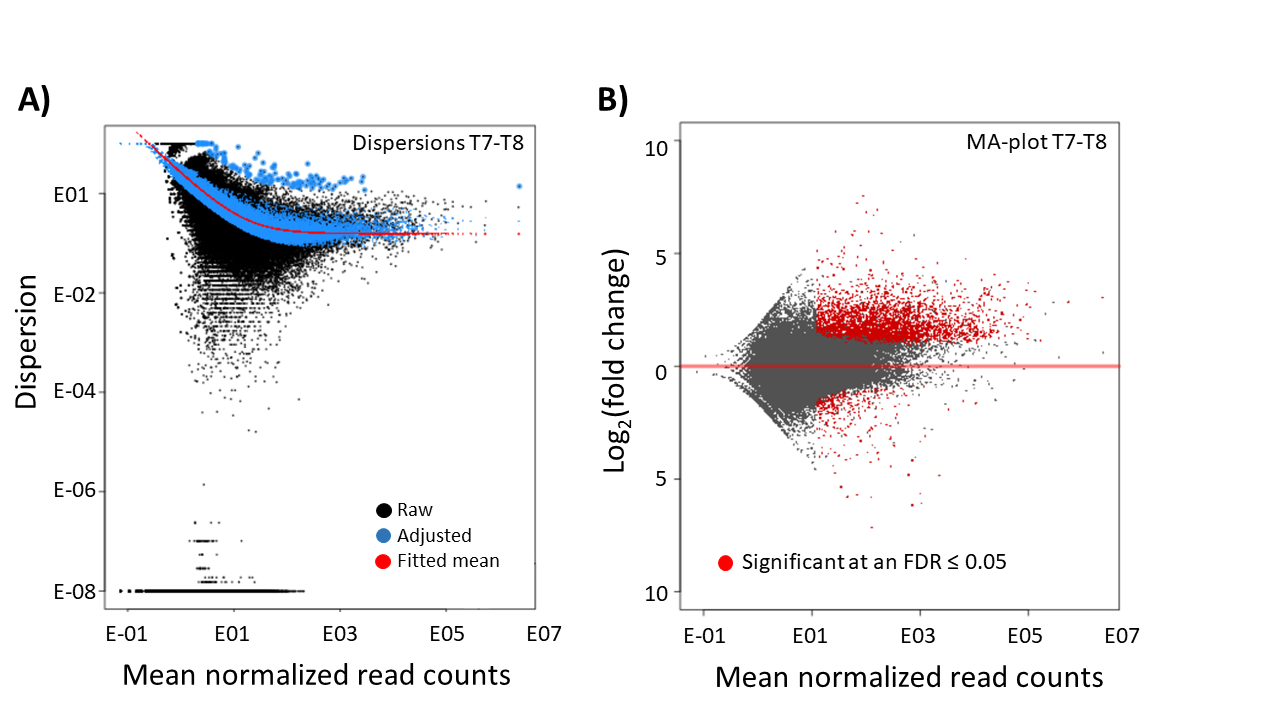


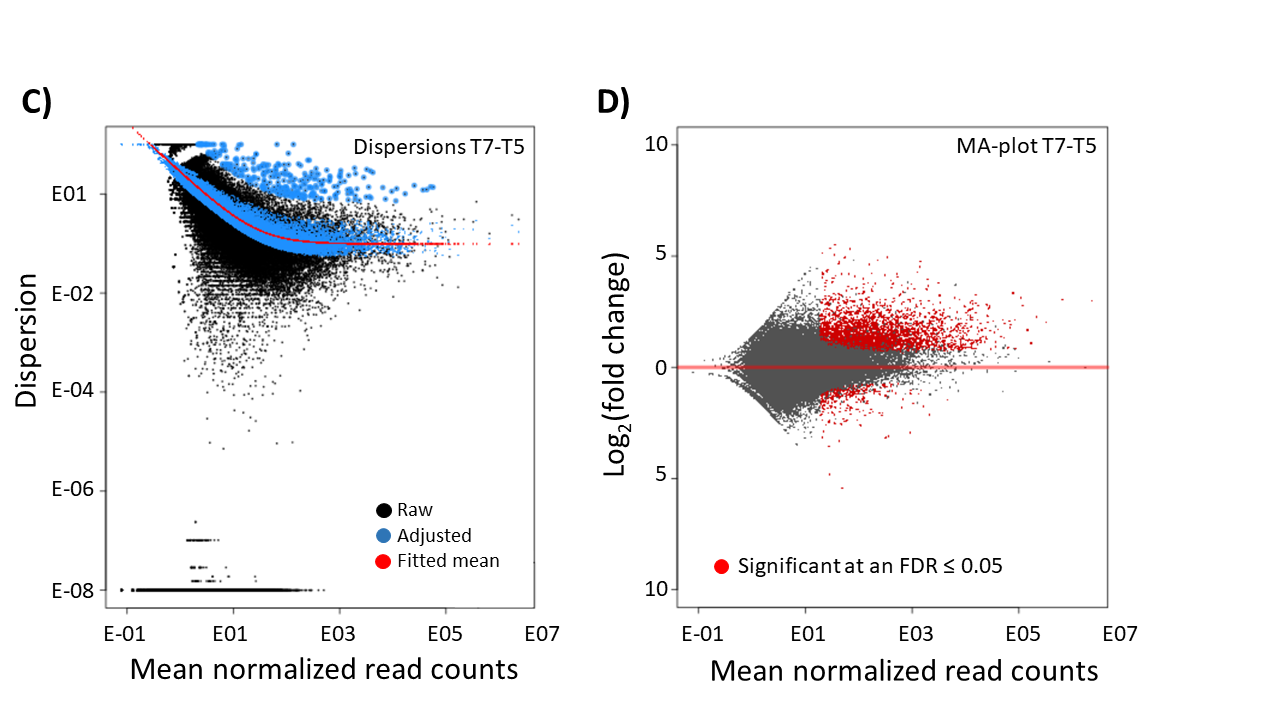

Supplement: Supplementary file 8 — Additional file 8: Supplementary Fig. S2. Dispersion of raw and DESeq2 adjusted read counts about empirical mean for comparisons of triplicate RNA-seq data within A) control maize (Cn; Treatment T7) and Cry3Bb1 maize (VT3; T8), and C) control maize (Cn; T7) and Gpp34/Tpp35Ab1 maize (Hx; T5). MA-plots of Log2 transformed fold-change estimates and normalized mean read count are shown for B) control maize (Cn; Treatment T7) and Cry3Bb1 maize (VT3; T8), and D) control maize (Cn; T7) and Gpp34/Tpp35Ab1 maize (Hx; T5), with datapoints (change in transcript read counts) surpassing a Benjamini and Hochberg (1995) adjusted false discovery rate (FDR) of ≤0.05 shown in red. [file 12864_2021_7932_MOESM8_ESM.docx]
