# Supplementary material for: Up-regulation of apoptotic- and cell survival-related gene pathways following exposures of western corn rootworm to B. thuringiensis crystalline pesticidal proteins in transgenic maize roots
Source: BMC Genomics. 2021 Sep 4;22:639. doi: 10.1186/s12864-021-07932-4 (PMC8418000; doi:10.1186/s12864-021-07932-4)

**Supplementary Figure S3:** Gene Ontology (GO) terms enriched among transcripts differentially expressed in *Heterorhabditis bacteriophora* (Hb) and *Metarhizium anisopliae* (Ma) treatments that were shared with treatments **A)** Cry3Bb1 and **B)** Gpp34/Tpp35Ab1. Significantly overrepresented GO terms are shown within categories biological process (BP) and cellular component (CC) (FDR ≤ 1.0E^-5^) and molecular function (MF) at level 2 (FDR ≤ 1.0E^-7^; grey bars). Categories listed by GO ID and GO term. Number of transcripts encoding each PFAM domain within each functional category are indicated (black bars).

1. **B)**


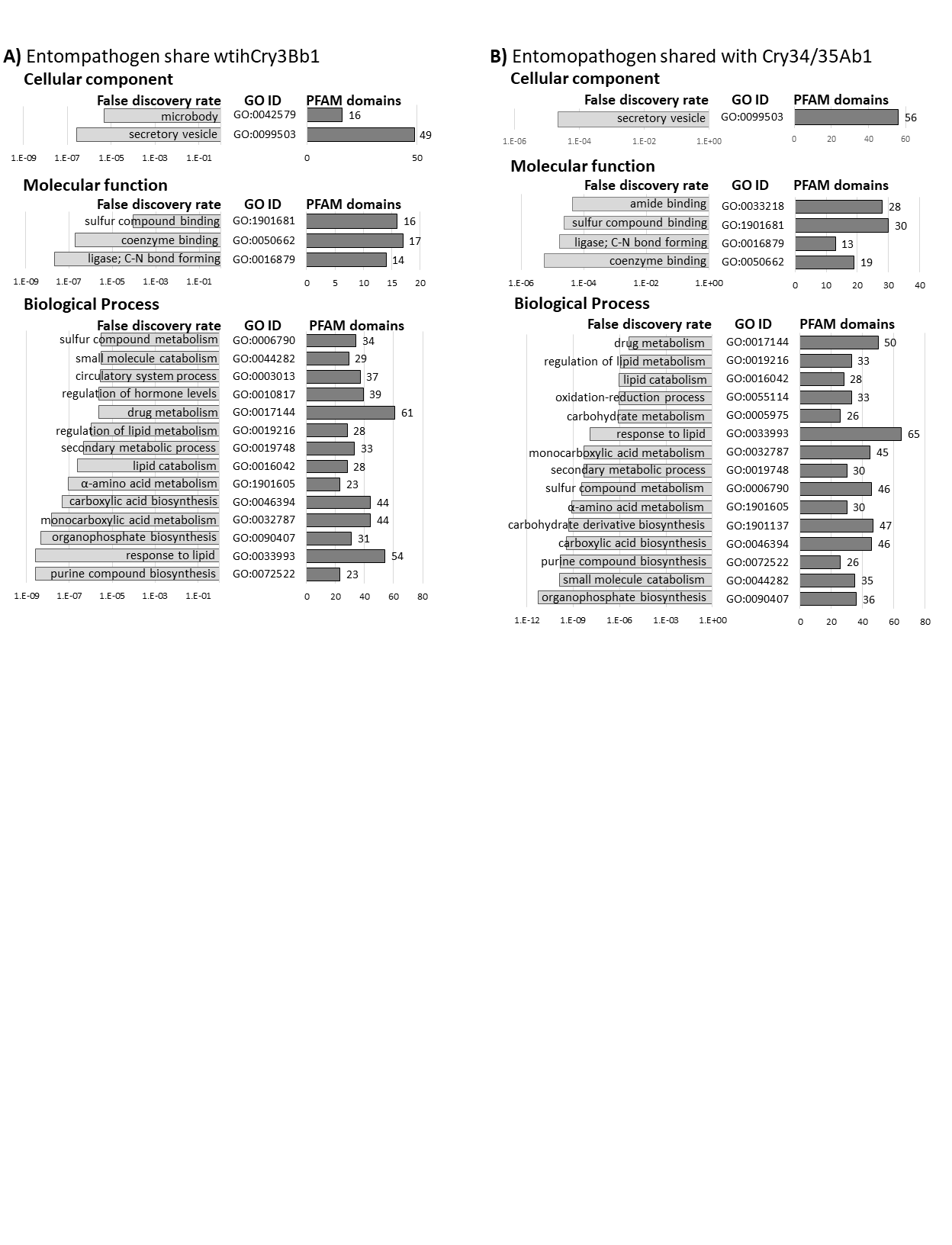

Supplement: Supplementary file 11 — Additional file 11: Supplementary Fig. S3. Gene Ontology (GO) terms enriched among transcripts differentially expressed in Heterorhabditis bacteriophora (Hb) and Metarhizium anisopliae (Ma) treatments that were shared with treatments A) Cry3Bb1 and B) Gpp34/Tpp35Ab1. Significantly overrepresented GO terms are shown within categories biological process (BP) and cellular component (CC) (FDR ≤ 1.0E− 5) and molecular function (MF) at level 2 (FDR ≤ 1.0E− 7; grey bars). Categories listed by GO ID and GO term. Number of transcripts encoding each PFAM domain within each functional category are indicated (black bars). [file 12864_2021_7932_MOESM11_ESM.docx]
