# Supplementary material for: Up-regulation of apoptotic- and cell survival-related gene pathways following exposures of western corn rootworm to B. thuringiensis crystalline pesticidal proteins in transgenic maize roots
Source: BMC Genomics. 2021 Sep 4;22:639. doi: 10.1186/s12864-021-07932-4 (PMC8418000; doi:10.1186/s12864-021-07932-4)
Supplement: Supplementary file 13 — Additional file 13: Supplementary Fig. S4. Alignment of partial enzymatic domain sequences from Drosophila melanogaster caspases DRONC, DRED, DAMM, STRICA, DCP-1, and DRICE (accessions in footnotes) with putative Diabrotica virgifera virgifera orthologs from the reference transcriptome assembly (DIAVI02NNNN) in this study and RefSeq gene models (XP_0281NNNNN) from the D. v. virgifera genome assembly Dvir_v2.0 (GCA_003013835.2; GenBank accession PXJM00000000.2). Conserved residues are highlighted grey, with those involved in binding and catalysis in black and yellow, respectively. [file 12864_2021_7932_MOESM13_ESM.docx]

**Supplementary Figure S4:** Alignment of partial enzymatic domain sequences from *Drosophila melanogaster* caspases DRONC, DRED, DAMM, STRICA, DCP-1, and DRICE (accessions in footnotes) with putative *Diabrotica virgifera virgifera* orthologs from the reference transcriptome assembly (DIAVI02NNNN) in this study and RefSeq gene models (XP_0281NNNNN) from the *D. v. virgifera* genome assembly Dvir_v2.0 (GCA_003013835.2; GenBank accession PXJM00000000.2). Conserved residues are highlighted grey, with those involved in binding and catalysis in black and yellow, respectively.

DRONC NRRRIGAEKDSKSLIHLFQELNFTI-FP---YGNVNQDQFFKLLTMVTSSSYVQNTECFVMVLMTHGNSV 56

XP_028140498.1 FPYRNGALVDDANLKALFEQMGGWDLDC---YHNKTALEMIMLIQKFAG-EGKPDYDICCMIIMSHGGEM 56

XP_028140499.1 FPYRNGALVDDANLKALFEQMGGWDLDC---YHNKTALEMIMLIQKFAG-EGKPDYDICCMIIMSHGGEM 56

DIAVI012702 NDERKGAELDEKNLKTLFRNMGF-DVYV---HRNQKLNEMKSKLNSYRQSRDVGRGDILIVIVMSHGNNA 56

XP_028134407.1 NDERKGAELDEKNLKTLFRNMGF-DVYV---HRNQKLNEMKSKLNSYRQSRDVGRGDILIVIVMSHGNNA 56

DRED LRRRDGTDVDKERLIEVFSSMGYNV-EA---YDNVDHMGIIERIRSACD--RSLVRDSLVVFILSHGFE- 54

XP_028130548.1 LEVREGTNKDRDNLINTFIRRGFKNIIV---ANNLNHIDMLEKIKDTVN--KTTNESSLFVCILSHGNE- 55

DIAVI055333 --------------------------------NNLNHIDMLEKIKDTVN--KTTNESSLFVCILSHGNE- 26

DAMM QLNRKGSSNDVNALRKTFESLKCRV-EV---ISNPALPDVKNKVKEWSA-KRFTQDAGFVLFILSHGDRK 55

STRICA NEFRKGSAQDVKVLRATFEQLKCKV-EV---ITDATLVTIKKTVRMLQT-KDFEDKSALVLVILSHGTRH 55

DIAVI022989 LGTRRGTRRDVNELTTCLQRLGFNI-DKENILTDKTTEEVEQVLEKASQ-IDFTNYNCLILFFLSHGEEL 58

XP_028132548.1 LGTRRGTRRDVNELTTCLQRLGFNI-DKENILTDKTTEEVEQVLEKASQ-IDFTNYNCLILFFLSHGEEL 58

DECAY QKQRVGTERDRDDMEATLQGFGFDV-RT---FDDLTFSEINDTLKEVAR-EDHSQNDCFVLAVMSHGTE- 55

DIAVI027204 VSTRDGTDLDRDALQNVLQELTFDV-LV---YDDLTLEKILEVLSVVSK-MNHNECECLIIAVMSHGDE- 55

XP_028144295.1 VSTRDGTDLDRDALQNVLQELTFDV-LV---YDDLTLEKILEVLSVVSK-MNHNECECLIIAVMSHGDE- 55

DCP-1 LKSRTGTNVDAQELKKAFENLGFAV-SV---HKDCKLRDILKHVGKAAE-LDHTDNDCLAVAILSHGEH- 55

DRICE LKSRTGTNVDAQELKKAFENLGFAV-SV---HKDCKLRDILKHVGKAAE-LDHTDNDCLAVAILSHGEH- 55

XP_028152387.1 LKPRNGTNEDCKNLKECLIVLGFDV-HV---FKDLNYRDIEQHVRETAQ-ADHNKHDCLLISVLSHGEM- 55

DIAVI017124 LKPRNGTNEDCKNLKECLIVLGFDV-HV---FKDLNYRDIEQHVRETAQ-ADHNKHDCLLISVLSHGEM- 55

XP_028154451.1 ------------------------------------MFTKNYILFSAAK-IDHNKYDCLLISVLSHGDL- 23

XP_028152860.1 LLTRRGTNEDCRNLKECLIALGFDV-QV---FKDLDNRDIEQHIDDTAK-IDHNKYDCLLISVLSHGDL- 55

DIAVI015400 LLTRRGTNEDCRNLKECLIALGFDV-QV---FKDLDNRDIEQHIDDTAK-IDHNKYDCLLISVLSHGDL- 55

DIAVI015965 LLTRRGTNEDCRNLKECLIALGFDV-QV---FKDLDNRDIEQHIDDTAK-IDHNKYDCLLISVLSHGDL- 55

DRONC EGKEKVEF-------CDGSVVDMQKIKDHFQTAKCPYLVNKPKVLMFPFCRGDEYDLG-HPKNQGNLMEP 109

XP_028140498.1 NNDTYIYG----IDEEQQYELSITKITKFFTNENSKSWRGKPKMLFFPICRGADNQLP-IKHEYRTET-- 112

XP_028140499.1 NNDTYIYG----IDEEQQYELSITKITKFFTNENSKSWRGKPKMLFFPICRGADNQLP-IKHEYRTET-- 112

DIAVI012702 RGSEEIPGGFTKIQTSDDKYLNVDEVLSEFTNDRCAPMKGKPKIFIFQCCRGHREELQ-IDAV------- 116

XP_028134407.1 RGSEEIPGGFTKIQTSDDKYLNVDEVLSEFTNDRCAPMKGKPKIFIFQCCRGHREELQ-IDAV------ 116

DRED ---EAVYA-------SNSIAMKITDIEDLLC--SYDTLYYKPKLLIIQACQEKLVHKK-KPNELF----- 101

XP_028130548.1 --GVVYG-------CNSCHVKVSKIQEIMS----NKNFNQKPKVLILQSCQGRECLKV-DSKDDDD---- 101

DIAVI055333 --GVVYG-------CNSCHVKVSKIQEIMS----NKNFNQKPKVLILQSCQGRECLKV-DSKDDDD---- 72

DAMM ---EKILA-------CDHREYHLDDD-VLFPLFRNPTLSGKPKILIVQACKGPLRADA------------ 104

STRICA ---DQIAA--------KDDDYSLDDD-VVFPILRNRTLKDKPKLIFVQACKGDCQLGG------------ 103

DIAVI022989 ---NFLHT--------KDGQILAKDIWLKFK--DSPGLNGKPKMFVFQACKGENFTTVGNELPKSAVL-- 105

XP_028132548.1 ---NFLHT--------KDGQILAKDIWLKFK--DSPGLNGKPKMFVFQACKGENFTTVGNELPKSAVL-- 105

DECAY ---GKVYA--------KDMSYPVERLWNPFLGDNCKTLKNKPKLFFIQACRGANLEKA-VEFSSFAVMT- 103

DIAVI027204 ---GIVYA--------RDQAYPTKKLWSYFTPIRCPSLAGKPKMFFIQACRGSESDPG--QVVTYVE--- 103

XP_028144295.1 ---GIVYA--------RDQAYPTKKLWSYFTPIRCPSLAGKPKMFFIQACRGSESDPG-QVVTYVE---- 103

DCP-1 ---GYLYA--------KDTQYKLDNIWHYFTATFCPSLAGKPKLFFIQACQGDRLDGG-ITLEKGV---- 103

DRICE ---GYLYA--------KDTQYKLDNIWHYFTATFCPSLAGKPKLFFIQACQGDRLDGG-ITLEKGV---- 103

XP_028152387.1 ---GIIYA--------KDTPYKPDNLWASFTADRCPSLAGKPKIFFLQACQGDKLDGG-VSLSR------ 103

DIAVI017124 ---GIIYA--------KDTPYKPDNLWASFTADRCPSLAGKPKIFFLQACQGDKLDGG-VSLSR------ 103

XP_028154451.1 ---GIVHA--------KDGPYKPETLWTPFTADKCPSLAGKPKIFFLQACRGRQFDSG-IILKK------ 71

XP_028152860.1 ---GIVHA--------KDGPYKPETLWTPFTADKCPSLAGKPKIFFLQACRGRQFDSG-IILKK------ 103

DIAVI015400 ---GIVHA--------KDGPYKPETLWTPFTADKCPSLAGKPKIFFLQACRGRQFDSG-IILKK------ 103

DIAVI015965 ---GIVHA--------KDGPYKPETLWTPFTADKCPSLAGKPKIFFLQACRGRQFDSG-IILKK------ 103

DRONC VYTAQEEKWPDTQTEGIPSPSTNVPSLADTLVCYAN-----TPGYVTHRDLDTGSWY 164

XP_028140498.1 -------DSAFKNVPANTPTVANLRAEEDMLVGFSTAMVFDVLGYKAHRDPYLGSWY 162

XP_028140499.1 -------DSAFKNVPANTPTVANLRAEEDMLVGFSTA-----MGYKAHRDPYLGSWY 159

DIAVI012702 ------------------PFTNIVKKHADMLIAFST-----VPGFSSIRAPQEGSWY 146

XP_028134407.1 ------------------PFTNIVKKHADMLIAFST-----VPGFSSIRAPQEGSWY 146

DRED ---------------RIDVTTVSPDQHIDMLRAMST-----VNGYAALRHTQTGSWF 136

XP_028130548.1 --------------SKSLTTDGGGTTYRDMLTFWAT-----FPGYAAIRNKQTGTWF 138

DIAVI055333 --------------SKSLTTDGGGTTYRDMLTLWAT-----FPGYAAIRNKQTGTWF 109

DAMM -----------K-------KM----NNEPYIKCYSC-----SEGYLSYRNENHGSVF 126

STRICA -----------FMTDAAQPNG----SPNEILKCYST-----YEGFVSFRTE-DGTPF 132

DIAVI022989 -----------TPSS----TFSPDYLYDDLLIVHSS-----TEGNCAFRNQYTGSWF 144

XP_028132548.1 -----------TPSS----TFSPDYLYDDLLIVHSS-----TEGNCAFRNQYTGSWF 144

DECAY ------RELVPEPAAAVQPITYAIPSTADILVFYST-----FDKFFSFRNVDDGSWF 151

DIAVI027204 -----------KDSRPTSSKTYTIPIMADIIVMYAT-----VEEYYAWRDPATGSYF 143

XP_028144295.1 -----------KDSRPASSKTYTIPIMADIIVMYAT-----VEEYYAWRDPDTGSYF 143

DCP-1 -----------TETDGESSTSYKIPIHADFLFSYST-----IPGYFSWRNINNGSWY 143

DRICE -----------TETDGESSTSYKIPIHADFLFSYST-----IPGYFSWRNINNGSWY 143

XP_028152387.1 -----------TETDGEIHNTYKIPVQADFLIVYST-----VKGYYSWRNTTKGSWF 141

DIAVI017124 -----------TETDGEIHNTYKIPVQADFLIVYST-----VKGYYSWRNTTKGSWF 141

XP_028154451.1 -----------TETDGEQ-TSYKIPVEADFLIVYST-----AIGYFSWRNPVKGSWF 108

XP_028152860.1 -----------TETDGEQ-TSYKIPVEADFLIVYST-----AIGYFSWRNPVKGSWF 140

DIAVI015400 -----------TETDGEQ-TSYKIPVEADFLIVYST-----AIGYFSWRNPVKGSWF 140

DIAVI015965 -----------TETDGEQ-TSYKIPVEADFLIVYST-----AIG----------SWF 140

*Drosophila* protein (Flybase ID; annotation symbol): DRONC, death regulator Nedd2-like caspase (FBgn0026404 CG8091); DRED, Death related ced-3/Nedd2-like caspase (FBgn0020381; CG7486), DAMM (FBgn0033659; CG18188), STRICA, Ser/Thr-rich caspase (FBgn0033051; CG7863), DECAY, Death executioner caspase related to Apopain/Yama (FBgn0028381; CG14902), DCP-1, death caspase 1 (FBgn0010501, CG5370), and DRICE, death related ICE-like caspase (FBgn0019972; CG7788).
