# Supplementary material for: Up-regulation of apoptotic- and cell survival-related gene pathways following exposures of western corn rootworm to B. thuringiensis crystalline pesticidal proteins in transgenic maize roots
Source: BMC Genomics. 2021 Sep 4;22:639. doi: 10.1186/s12864-021-07932-4 (PMC8418000; doi:10.1186/s12864-021-07932-4)
Supplement: Supplementary file 14 — Additional file 14: Supplementary Fig. S5. Structural annotation of inhibitor of apoptosis proteins (IAPs) encoded by transcripts differentially expressed following Diabrotica virgifera virgifera exposure to Cry3Bb1 or Gpp34/Tpp35Ab1. Annotations based on structure defined by Hay et al. (2000). [file 12864_2021_7932_MOESM14_ESM.docx]

**Supplementary Figure S5:** Structural annotation of inhibitor of apoptosis proteins (IAPs) encoded by transcripts differentially expressed following *Diabrotica virgifera virgifera* exposure to Cry3Bb1 or Gpp34/Tpp35Ab1. Annotations based on structure defined by Hay et al. (2000).

**A)** Annotation of *D. v. virgifera* IAP1 (DvvIAP1) isoforms encoded by transcripts DIAVI011972 (XP_028141650.1 isoform X1; DvvIAP1_X1) and DIAVI007715 (XP_028141655.1 isoform X5; DvvIAP1_X5). Two baculovirus inhibition of apoptosis protein repeat domains (BIR1 and BIR2) and RING finder domains characteristic of IAP family 1 proteins are underlined, and cysteine and histidine residues involved in tetrahedral coordination of Zn+ required to form the zinc-finger-like protein structure are highlighted grey. Table shows results sequence query of the conserved domain database (Marchler-Bauer A et al. 2017).

DIAVI011972: DvvIAP1_X1 significantly up-regulated in *D. v. virgifera* larvae exposed to the *Bacillus thuringiensis* toxin Cry34/35Ab1.

1-MTHIVKWLWTKLFHGTKMAVVQSNYIQNIPSFGCVDQPDNGSKTTRESLVEVSSSRPRQEDYSVYENRLA

71-SFTNWPNTQVSRESLARAGFIYTGQDDIVICPICKIEGYRWVSGDNPMDDHRVWNPNCPFLNRRDNIEHD

141-HSVGSRDTCGLFGIELLPNSVPEDNTSNLQKLGIQPGTGPQNQDKITLESRLATFQGWPKSIKQRPSELA

211-EAGFYYTGAGDQTVCFYCGGGLKDWDEGDDPWEQHALWFSKCVFLNLKKGKEFIDQVKRKADPQFSIPGP

351-SGTQAKEEPTATESSSDKQSETVKTKSDRESFATDTTLCKICFKNELGVVFLPCGHIVACVDCAAALKTC

421-AVCRKPLEATVRAFLS*

Results of querying the conserved domain database with the full translated DIAVI011972 protein sequence.

| Feature | Accession | Description | Interval | E-value |
| --- | --- | --- | --- | --- |
| BIR1 | [cd00022](https://www.ncbi.nlm.nih.gov/Structure/cdd/cddsrv.cgi?ascbin=8&maxaln=10&seltype=2&uid=cd00022) | Baculoviral inhibition of apoptosis protein repeat domain 1 | 66-132 | 5.75e-34 |
| BIR2 | [cd00022](https://www.ncbi.nlm.nih.gov/Structure/cdd/cddsrv.cgi?ascbin=8&maxaln=10&seltype=2&uid=cd00022) | Baculoviral inhibition of apoptosis protein repeat domain 2 | 189-257 | 3.08e-25 |
| RING | [cl17238](https://www.ncbi.nlm.nih.gov/Structure/cdd/cddsrv.cgi?ascbin=8&maxaln=10&seltype=2&uid=cl17238) | The superfamily of Really interesting new gene (RING) finger domain and U-box domain | 315-366 | 5.29e-18 |

DIAVI007715: DvvIAP1_X5 significantly up-regulated in *D. v. virgifera* larvae exposed to the *Bacillus thuringiensis* toxin Cry3Bb1.

1-MAVVQSNYIQNIPSFGCVDQPDNGSKTTRESLVEVSSSRPRQEDYSVYENRLASFTNWPNTQVSRESLAR

71-AGFIYTGQDDIVICPICKIEGYRWVSGDNPMDDHRVWNPNCPFLNRRDNIEHDHSVGSRDTCGLFGIELL

141-PNSVPEDNTSNLQKLGIQPGTGPQNQDKITLESRLATFQGWPKSIKQRPSELAEAGFYYTGAGDQTVCFY

211-CGGGLKDWDEGDDPWEQHALWFSKCVFLNLKKGKEFIDQVKRKADPQFSIPGPSGTQAKEEPTATESSSD

351-KQSETVKTKSDRESFATDTTLCKICFKNELGVVFLPCGHIVACVDCAAALKTCAVCRKPLEATVRAFLS*

Results of querying the conserved domain database with the full translated DIAVI007715 protein sequence.

| Feature | Accession | Description | Interval | E-value |
| --- | --- | --- | --- | --- |
| BIR1 | [cd00022](https://www.ncbi.nlm.nih.gov/Structure/cdd/cddsrv.cgi?ascbin=8&maxaln=10&seltype=2&uid=cd00022) | Baculoviral inhibition of apoptosis protein repeat domain 1 | 49-115 | 1.99e-25 |
| BIR2 | [cd00022](https://www.ncbi.nlm.nih.gov/Structure/cdd/cddsrv.cgi?ascbin=8&maxaln=10&seltype=2&uid=cd00022) | Baculoviral inhibition of apoptosis protein repeat domain 2 | 172-240 | 3.54e-34 |
| RING | [cl17238](https://www.ncbi.nlm.nih.gov/Structure/cdd/cddsrv.cgi?ascbin=8&maxaln=10&seltype=2&uid=cl17238) | The superfamily of Really interesting new gene (RING) finger domain and U-box domain | 298-349 | 7.45e-18 |

**B)** Annotation of *D. v. virgifera* IAP2 (DvvIAP2) encoded by transcript DIAVI011430. Three baculovirus inhibition of apoptosis protein repeat domains (BIR1, BIR2 and BIR3) and RING finder domains characteristic of IAP family 2 proteins are underlined, and cysteine and histidine residues involved in tetrahedral coordination of Zn+ required to form the zinc-finger-like protein structure are highlighted grey.

DIAVI011430: DvvIAP2 not significantly up-regulated in *D. v. virgifera* larvae exposed to the *Bacillus thuringiensis* toxin Cry3Bb1 or Cry34/35Ab1.

1-MHQEINRLQTFSEWPSDAQVSPQRIAKAGFFATKQGLEVECFACHAKISEWNYGDQVMTRHIALNRDCPF

71-VLNPSTSGNVPITSSRVPSTSINMYRSSETRLASFENWPAADIVTPDSLVQAGFYYLKEGDNTQCAFCKG

141-VVRAWEVGDDPDTEHQRHFPNCPFVMAVINPRLQARRGSNDRNNPENNQIVKDSFPNINVVGTEQNLGEL

211-GVQAHRGPKKSNFATVEARLRSYVGWSSDLIQTPEVLAEAGFYYEGMGDQVRCFHCDGGLRTWDPHDDPW

281-TEHARWFPNCSFVKLVKGQDFVTACTIGQTTDSSVRPSAQRIQTTRIRREVTEREIQSYLTSPQALAALS

351-IGLNVERVKRAIREKLEQTGRAYSQPDALVEAALNLQHEEEDPNSHEHYTPIDRSLRNVVCAAMEECIDR

421-QPEQVQQPEPAQQPEMDEAFEVSPTTAPDGTPQLHYQLVKTVSLEEENRILKEARLCKICMDSEVGIVFL

491-PCGHLATCVNCAPNLEDCPVCRSTIKATVRTFFS*

Results of querying the conserved domain database with the full translated DIAVI011430 protein sequence.

| Feature | Accession | Description | Interval | E-value |
| --- | --- | --- | --- | --- |
| BIR1 | [cd00022](https://www.ncbi.nlm.nih.gov/Structure/cdd/cddsrv.cgi?ascbin=8&maxaln=10&seltype=2&uid=cd00022) | Baculoviral inhibition of apoptosis protein repeat domain; Found in inhibitors of apoptosis ... | 5-73 | 9.93e-24 |
| BIR2 | [cd00022](https://www.ncbi.nlm.nih.gov/Structure/cdd/cddsrv.cgi?ascbin=8&maxaln=10&seltype=2&uid=cd00022) | Baculoviral inhibition of apoptosis protein repeat domain; Found in inhibitors of apoptosis ... | 99-167 | 4.92e-30 |
| BIR3 | [cd00022](https://www.ncbi.nlm.nih.gov/Structure/cdd/cddsrv.cgi?ascbin=8&maxaln=10&seltype=2&uid=cd00022) | Baculoviral inhibition of apoptosis protein repeat domain; Found in inhibitors of apoptosis ... | 227-295 | 7.23e-32 |
| UBA_IAPs | [cd14321](https://www.ncbi.nlm.nih.gov/Structure/cdd/cddsrv.cgi?ascbin=8&maxaln=10&seltype=2&uid=cd14321) | ubiquitin-association(UBA) domain found in inhibitor of apoptosis proteins (IAPs); IAPs are frequently overexpressed ... | 343-386 | 4.56e-11 |
| RING-HC_BIRC2_3_7 | [cd16713](https://www.ncbi.nlm.nih.gov/Structure/cdd/cddsrv.cgi?ascbin=8&maxaln=10&seltype=2&uid=cd16713) | RING finger, HC subclass, found in apoptosis protein c-IAP1, c-IAP2, livin, and similar ... | 471-524 | 1.10e-27 |

**C)** Multiple protein alignment of baculoviral inhibition of apoptosis protein repeat (BIR) domains from *Drosophila melanogaster* inhibitor of apoptosis protein 1 and 2 [DIAP1 (AAF49548.1) and DIAP2 (AAF58095.1)] with putative *Diabrotica virgifera virgifera* IAP1 orthologs encoded by isoforms X1 (DvvIAP1_X1) and X5 (DvvIAP1_X5), and IAP2 (DvvIAP2). Conserved cysteine and histidine residues involved in tetrahedral coordination of Zn+ required to form the zinc-finger-like structure are highlighted yellow, and remaining conserved residues with 100% conservation are in dark grey and ≥50% in light grey.

Two BIR repeats from DIAP1 orthologs

DIAP1A_BIR1 -ETRLKTFTDWPLDWLDK-RQLAQTGMYFTHAGDKVKCFFCGVEIGCWEQEDQPVPEHQRWSPNCPLLRR 68

DvvAIP1X1_BIR1 -ENRLASFTNWPNTQVSR-ESLARAGFIYTGQDDIVICPICKIEGYRWVSGDNPMDDHRVWNPNCPFLN- 68

DvvAIP1X5_BIR1 -ENRLASFTNWPNTQVSR-ESLARAGFIYTGQDDIVICPICKIEGYRWVSGDNPMDDHRVWNPNCPFLN- 68

DIAP1A_BIR2 ETARLRTFEAWPRNLKQKPHQLAEAGFFYTGVGDRVRCFSCGGGLMDWNDNDEPWEQHALWLSQCRFVKL 70

DvvAIP1X1_BIR2 -ESRLATFQGWPKSIKQRPSELAEAGFYYTGAGDQTVCFYCGGGLKDWDEGDDPWEQHALWFSKCVFLNL 69

DvvAIP1X5_BIR2 -ESRLATFQGWPKSIKQRPSELAEAGFYYTGAGDQTVCFYCGGGLKDWDEGDDPWEQHALWFSKCVFLNL 69

Three BIR repeats from DIAP2 orthologs

DIAP2A_BIR1 -SVRLATFGEWPLNAPVSAEDLVANGFFATGNWLEAECHFCHVRIDRWEYGDQVAERHRRSSPICSMVLA 69

DvvAIP2_BIR1 -INRLQTFSEWPSDAQVSPQRIAKAGFFATKQGLEVECFACHAKISEWNYGDQVMTRHIALNRDCPFVLN 68

DIAP2A_BIR2 -EANRLVTFKDWPNPNITPQALAKAGFYYLNRLDHVKCVWCNGVIAKWEKNDNAFEEHKRFFPQCPRVQM 69

DvvAIP2_BIR2 -ETRLASFENWPAADIVTPDSLVQAGFYYLKEGDNTQCAFCKGVVRAWEVGDDPDTEHQRHFPNCPFVMA 69

DIAP2A_BIR3 -EARLRTFTDWPISNIQPASALAQAGLYYQKIGDQVRCFHCNIGLRSWQKEDEPWFEHAKWSPKCQFVLL 69

DvvAIP2_BIR3 -EARLRSYVGWSSDLIQTPEVLAEAGFYYEGMGDQVRCFHCDGGLRTWDPHDDPWTEHARWFPNCSFVKL 69

>DvvAIP1X1_BIR1

ENRLASFTNWPNTQVSRESLARAGFIYTGQDDIVICPICKIEGYRWVSGDNPMDDHRVWNPNCPFLN

>DvvAIP1X1_BIR2

ESRLATFQGWPKSIKQRPSELAEAGFYYTGAGDQTVCFYCGGGLKDWDEGDDPWEQHALWFSKCVFLNL

>DvvAIP1X5_BIR1

ENRLASFTNWPNTQVSRESLARAGFIYTGQDDIVICPICKIEGYRWVSGDNPMDDHRVWNPNCPFLN

>DvvAIP1X5_BIR2

ESRLATFQGWPKSIKQRPSELAEAGFYYTGAGDQTVCFYCGGGLKDWDEGDDPWEQHALWFSKCVFLNL

>DIAP1A_BIR1

ETRLKTFTDWPLDWLDKRQLAQTGMYFTHAGDKVKCFFCGVEIGCWEQEDQPVPEHQRWSPNCPLLRR

>DIAP1A_BIR2

ETARLRTFEAWPRNLKQKPHQLAEAGFFYTGVGDRVRCFSCGGGLMDWNDNDEPWEQHALWLSQCRFVKL

>DIAVI011430_DvvAIP2_BIR1

NRLQTFSEWPSDAQVSPQRIAKAGFFATKQGLEVECFACHAKISEWNYGDQVMTRHIALNRDCPFVLN

>DIAVI011430_DvvAIP2_BIR2

ETRLASFENWPAADIVTPDSLVQAGFYYLKEGDNTQCAFCKGVVRAWEVGDDPDTEHQRHFPNCPFVMAV

>DIAVI011430_DvvAIP2_BIR3

ARLRSYVGWSSDLIQTPEVLAEAGFYYEGMGDQVRCFHCDGGLRTWDPHDDPWTEHARWFPNCSFVKL

>DIAP2A_BIR1

RLATFGEWPLNAPVSAEDLVANGFFATGNWLEAECHFCHVRIDRWEYGDQVAERHRRSSPICSMVLA

>DIAP2A_BIR2

EANRLVTFKDWPNPNITPQALAKAGFYYLNRLDHVKCVWCNGVIAKWEKNDNAFEEHKRFFPQCPRVQM

>DIAP2A_BIR3

ARLRTFTDWPISNIQPASALAQAGLYYQKIGDQVRCFHCNIGLRSWQKEDEPWFEHAKWSPKCQFVLL

>AAF49548.1_DIAP1A

MASVVADLPSYGPIAFDQVDNNTNATQLFKNNINKTRMNDLNREETRLKTFTDWPLDWLDKRQLAQTGMY

FTHAGDKVKCFFCGVEIGCWEQEDQPVPEHQRWSPNCPLLRRRTTNNVPINAEALDRILPPISYDICGAN

DSTLEMREHAYAEGVIPMSQLIQSIGMNAVNAAGSVTGTAAPQPRVTVATHASTATQATGDVQPETCRPS

AASGNYFPQYPEYAIETARLRTFEAWPRNLKQKPHQLAEAGFFYTGVGDRVRCFSCGGGLMDWNDNDEPW

EQHALWLSQCRFVKLMKGQLYIDTVAAKPVLAEEKEESSSIGGVAVASTQASEEEQQTSLSSEEAVSGDV

APSVAPTAATRIFNKIVEATAVATPSTNSSGSTSIPEEKLCKICYGAEYNTAFLPCGHVVACAKCASSVT

KCPLCRKPFTDVMRVYFS
