# Supplementary material for: Up-regulation of apoptotic- and cell survival-related gene pathways following exposures of western corn rootworm to B. thuringiensis crystalline pesticidal proteins in transgenic maize roots
Source: BMC Genomics. 2021 Sep 4;22:639. doi: 10.1186/s12864-021-07932-4 (PMC8418000; doi:10.1186/s12864-021-07932-4)
Supplement: Supplementary file 15 — Additional file 15: Supplementary Fig. S6. Multiple protein sequence alignment for B-cell-lymphoma protein 2 (Bcl-2)-associated X (BAX) inhibitor (BI) proteins (BI) and Lifeguard 4-like (LFG4) orthologs. [file 12864_2021_7932_MOESM15_ESM.docx]

**Supplementary Figure S6:** Multiple protein sequence alignment for B-cell-lymphoma protein 2 (Bcl-2)-associated X (BAX) inhibitor (BI) proteins (BI) and Lifeguard 4-like (LFG4) orthologs.

**A)** Alignment of orthologs for the putative BI-1 encoded by the transcript DIAVI026079 that is differentially expressed between Gpp34/Tpp35Ab1 exposed and unexposed *D. v. virgifera* (Dvv) larvae. Accession provided for *Anoplophora glabripennis* (AG), *Dendroctonus ponderosae* (Dp), *Drosophila melanogaster* (Dm), *Leptinotarsa decemlineata* (Ld), and *Tribolium castaneum* (Tc). Isoforms are designated as A or B following accessions for Dm. Residues with 100% conservation are highlighted dark grey and those ≥50% are in light grey. Transmembrane regions are double underlined for corresponding regions annotated within D. melanogaster UniProt record Q9VSH3 and putative TMRs from DIAVI026079 predicted by TMHMM 2.0.

DIAVI026079 --MATEYVRFSPNAFLNSFNAKLEQPVRQHLKNVYACLAMSTMSAALGAAVHLFTNIMQA 58

XP_028141533.1_Dvv --MATEYVRFSPNAFLNSFNAKLEQPVRQHLKNVYACLAMSTMSAALGAAVHLFTNIMQA 58

XP_971485.1_Tc -------MAPSVQTFLNSFSNSLEAPVRQHLKNVYACLAMSTMAAAIGASIHLFTNIIQA 53

XP_018561952.1_Ag ------MAAPSVQTFLNSFNNKLEEPVRQHLKNVYACLAMSTMAVAIGASVHIFTDFLQA 54

XP_023023598.1_Ld ------MAAASVQTFLNSFDAKLEQPVRAHLKNVYACLAMSTMAAAVGASVHLFTNILQA 54

XP_019762956.1_Dp ------MASVGINNFINSFNNRLEPPVKQHLKNVYACLAMSTLAAGVGGSIHLFTNLLQA 54

ERL83674.1_Dp_Hyp ---------------------------------------MSTLAAGVGGSIHLFTNLLQA 21

NP_648205.1_DmA MADTANYINDRFQTFMNGLGDRYEPYVREHLSKVYMVLGSTAAATAMGAMLQ-MRDFLDL 59

NP_729358.1_DMB MADTANYINDRFQTFMNGLGDRYEPYVREHLSKVYMVLGSTAAATAMGAMLQ-MRDFLDL 59

DIAVI026079 GFLSAIGALVFFFLLVNTPDDNGKSMTTRVGYLLGFTFLTGVGLGPLLEHVILVEPSIII 118

XP_028141533.1_Dvv GFLSAIGALVFFFLLVNTPDDNGKSMTTRVGYLLGFTFLTGVGLGPLLEHVILVEPSIII 118

XP_971485.1_Tc GFLSGIGALIFFGLLMATPDDNGKGLKMRIGYLLGFTTLTGVGMGPLLEHVIAVDPSIIV 113

XP_018561952.1_Ag GFLSAIGALVFFFLLMSTPDNNGKGLTLRVGYLLGFTFLTGVGMGPLLDFVIMVDPSIIV 114

XP_023023598.1_Ld GFISAIGALVCFYLLMSTPDDNGKAMTKRVGYLLGFSGLTGVGMGPLLEHVILVNPTIIV 114

XP_019762956.1_Dp GFLSGIGAIIFFFLLISTPDDNGKGMMKRVGYLLGFATLTGVGMGPLLEHVILVNPSIII 114

ERL83674.1_Dp_HyP GFLSGIGAIIFFFLLISTPDDNGKGMMKRVGYLLGFATLTGVGMGPLLEHVILVNPSIII 81

NP_648205.1_DmA GVLAAVATLVLV-LGLHFYKDDGKNYYTRLGMLYAFGFCSGQTLGPLLGYICSINPAIIL 118

NP_729358.1_DMB GVLAAVATLVLV-LGLHFYKDDGKNYYTRLGMLYAFGFCSGQTLGPLLGYICSINPAIIL 118

DIAVI026079 TAFVATSTVFVSFSAASMLSERGRWLYLGGTLMTMLTTLMVLSLANIFFGAMWIYQTQLY 178

XP_028141533.1_Dvv TAFVATSTVFVSFSAASMLSERGRWLYLGGTLMTMLTTLMVLSLANIFFGAMWIYQTQLY 178

XP_971485.1_Tc TALIGTAVVFVSFSVCSLLAERGKWLYLGGTLMSLLSTLMILSLANLFFGSSMLFQIQLY 173

XP_019762956.1_Dp TAFIATSVVFVSFSICAIFSERGKWLYLGGTLFTLLNSLMLMSLANILFGSTLLWNIQIY 174

ERL83674.1_Dp_HypP TAFIATSVVFVSFSICAIFSERGKWLYLGGTLFTLLNSLMLMSLANILFGSTLLWNIQIY 141

XP_023023598.1_Ld TAFFATSAVFVCFSICAMLSERGKWLYLGGTLMSMLTALMVLSFANLFFGAVWIYQTQLY 174

XP_018561952.1_Ag TAFIGTSAVFLCFSICALLSERGKWLYLGGTLMSIITILMLLSLANIFFGAMWVYQAQLY 174

NP_648205.1_DmA SALTGTFVTFISLSLSALLAEQGKYLYLGGMLVSVINTMALLSLFNMVFKSYFVQVTQLY 178

NP_729358.1_DMB SALTGTFVTFISLSLSALLAEQGKYLYLGGMLVSVINTMALLSLFNMVFKSYFVQVTQLY 178

DIAVI026079 LGLLAMCGFVLYDTQMIIEKRRLGSRDFVAHSLDLFVDFIGIFRRLLVILTQREQEAQRK 238

XP_028141533.1_Dvv LGLLAMCGFVLYDTQMIIEKRRLGSRDFVAHSLDLFVDFIGIFRRLLVILTQREQEAQRK 238

XP_971485.1_Tc LGLFAMCGFVLYDTQLIIEKRRLGSKDFVTHSLDLFVDFIGIFRRVLIILTQKEQESQKK 233

XP_019762956.1_Dp LGLFAMCGFVLYDTQAIIEKRRMGSKDFVAHSLDLFVDFIGVFKRLLIILTQKEQDQKKK 234

ERL83674.1_Dp_HypP LGLFAMCGFVLYDTQAIIEKRRMGSKDFVAHSLDLFVDFIGVFKRLLIILTQKEQDQKKK 201

XP_023023598.1_Ld LGLLAMCGFVLFDTQMIIEKRRAGSRDFVAHSLDLFVDFIGIFRRLLIILTQKEQDSRKK 234

XP_018561952.1_Ag VGLLAMCGFVLYDTQVIVEKRRMGSKDFVGHSLDLFIDFIGIFKRLLIILTQKEQNSRKK 234

NP_648205.1_DmA VGVFVMAAFIVYDTQNIVEKCRNGNRDVVQHALDLFFDVLSMFRRLLIILTQKEERKQNE 238

NP_729358.1_DMB VGVFVMAAFIVYDTQNIVEKCRNGNRDVVQHALDLFFDVLSMFRRLLIILTQKLKTFMV- 237

DIAVI026079 KRRD*-- 242

XP_028141533.1_Dvv KRRD--- 242

XP_971485.1_Tc RRN---- 236

XP_019762956.1_Dp KGNN--- 238

ERL83674.1_Dp_HypP KGNN--- 205

XP_023023598.1_Ld RRD---- 237

XP_018561952.1_Ag RRN---- 237

NP_648205.1_DmA RRQNKTK 245

NP_729358.1_DMB ------- 237

**B)** Alignment of orthologs for the putative LFG4 encoded by the transcript DIAVI029891 that is differentially expressed among Cry3Bb1 and Gpp34/Tpp35Ab1 exposed *D. v. virgifera* (Dvv) larvae compared to non-exposed cohorts. Isoforms are designated as A, B, C or D following accessions for Dm. Residues with 100% conservation are highlighted dark grey and those ≥50% are in light grey. Helical domains double underlined determined for *D. v. virgifera* proteins using TMHMM 2.0, and retrieved from Uniprot records for the orthologs from *D. melanogaster* (Dm; NP_610824.1 isoform A: Q6AWP0; NP_725236.1 isoform B: Q8T8W2; NP_001260927.1 isoform C), and *T. castaneum* (Tc; XP_969476.1: D6WLJ9). Abbreviations for species associated with corresponding accessions are provided above.

DIAVI029891_Dvv --------------------------------------MSQTVSLILNEDVEHGGKEYDE 22

XP_028155171.1_Dvv --------------------------------------MSQTVSLILNEDVEHGGKEYDE 22

XP_969476.1_Tc --------------------------------------MSSTVPLILEEDCERGGKDFDE 22

XP_018567206.1_Ag --------------------------------------MSQTIPLILEEDVEQGGKEYDE 22

XP_023024494.1_Lc --------------------------------------MSQTVPLILQEDIEQGGKEYED 22

XP_019762126.1_Dp MKDIYFEVSYNLGKHLLKFLWSKQSQFISIKVCEQEIKMSQSVPLILTEDVERGGKEYEE 60

NP_610824.1_DmA --------------------------------------------------MSSDNHFQYD 10

NP_725236.1_DmB ---------------------------------------------MYHYQQGDETGAYTD 15

NP_001260927.1_DmC ------------------------------------------------------------ 0

NP_001260928.1_DmD --------------------------------------------------MSSDNHFQYD 10

DIAVI029891_Dvv DDIENDFAYRNNVLNATKQIRLAFIRKVYGLLSMQILLTFIIASICLFTPPIKSFVHTND 82

XP_028155171.1_Dvv DDIENDFAYRNNVLNATKQIRLAFIRKVYGLLSMQILLTFIIASICLFTPPIKSFVHTND 82

XP_969476.1_Tc EGIENDFAYRNNVMQASKTIRLGFIRKVYGLLSMQLLLTIVVASIFMFTPQIKTFVHEND 82

XP_018567206.1_Ag -DIENDFAYRNNVAQATKSIRMAFIRKVYGLLTMQIFLTIVIASICMFTPPIKDFVHSND 81

XP_023024494.1_Lc -EIENDFAYRNNVWQATKSIRLAFIRKVYGLLTMQIFLTIVIAAVCMFTPPIRSFVQSND 81

XP_019762126.1_Dp NDIENDFAYRNNVAQATKSIRLAFLRKVYGLLTMQILLTVTIAAIFMFTPPIKVFVQTND 120

NP_610824.1_DmA AEADKSFAF------DDQSIRKGFIRKVYLILMCQLLITFGFVSVFTFSKASQEWVQKNP 64

NP_725236.1_DmB AEADKSFAF------DDQSIRKGFIRKVYLILMCQLLITFGFVSVFTFSKASQEWVQKNP 69

NP_001260927.1_DmC --------------------------------MCQLLITFGFVSVFTFSKASQEWVQKNP 28

NP_001260928.1_DmD AEADKSFAF------DDQSIRKGFIRKVYLILMCQLLITFGFVSVFTFSKASQEWVQKNP 64

DIAVI029891_Dvv ---WMMMFSFIASIALLVPLHIKRKESPTNFILLIAFTVVQAYTIGVIVTFYSKVVVLQA 139

XP_028155171.1_Dvv ---WMMMFSFIASIALLVPLHIKRKESPTNFILLIAFTVVQAYTIGVIVTFYSKVVVLQA 139

XP_969476.1_Tc ---WMLLVSFIPSIFLLIALIIKRRDTPANLILLAAFTVVEAYTVGVILTYYSQAVVLQA 139

XP_018567206.1_Ag ---WILMPTFLGSIALLLALHIKRRESPTNLILLAAFTIVQAYTIGVIVTFYSQAVVLQA 138

XP_023024494.1_Lc ---WMMLVTFFGSIALLLALHVKRRESPANFILLTAFTVVQAYTIGVIVTFYSQAVVLQA 138

XP_019762126.1_Dp ---WMMMISFFASIILLIPLHIKRRESPTNLILLAAFTIVQAYTIGVIVTFYSKAIVLEA 177

NP_610824.1_DmA ALFWIALAVLIVTMICMACCESVRRKTPLNFIFLFLFTVAESFLLGMVAGQFEADEVLMA 124

NP_725236.1_DmB ALFWIALAVLIVTMICMACCESVRRKTPLNFIFLFLFTVAESFLLGMVAGQFEADEVLMA 129

NP_001260927.1_DmC ALFWIALAVLIVTMICMACCESVRRKTPLNFIFLFLFTVAESFLLGMVAGQFEADEVLMA 88

NP_001260928.1_DmD ALFWIALAVLIVTMICMACCESVRRKTPLNFIFLFLFTVAESFLLGMVAGQFEADEVLMA 124

DIAVI029891_Dvv LVLTLVVLVALTAFTFQTKRDFSATHSALFAGLCILIVGGFMQIFIQSSILELGIGLGGA 199

XP_028155171.1_Dvv LVLTLVVLVALTAFTFQTKRDFSATHSALFAGLCILIVGGFMQIFIQSSILELGIGLGGA 199

XP_969476.1_Tc LLLTLVIVGSLTFYTFQTKRDFSAMYSGLFAGLGILIVGGFLQIFFHSSTFEIVISLGGA 199

XP_018567206.1_Ag LLLTFVVLAGLTLYTFQTKRDFSAIHSALFAGLCILIIGGFMQVFLQSTIFEIAIGFSGA 198

XP_023024494.1_Lc LLLTLVVLGGLTLYTFQTKRDFSAMHSGLFAGLIILIIGGSMQAFLQSSILEIAISLGGA 198

XP_019762126.1_Dp LLLTLLVLGGLTIYTFQSKHDFSAMHSGLFAGLLILIVGGFIQVFIQSPIFELLIGFGGA 237

NP_610824.1_DmA VGITAAVALGLTLFALQTKYDFTMCGGVLVACLVVFIIFGIIAIFIPGKVIGLVYASLGA 184

NP_725236.1_DmB VGITAAVALGLTLFALQTKYDFTMCGGVLVACLVVFIIFGIIAIFIPGKVIGLVYASLGA 189

NP_001260927.1_DmC VGITAAVALGLTLFALQTKYDFTMCGGVLVACLVVFIIFGIIAIFIPGKVIGLVYASLGA 148

NP_001260928.1_DmD VGITAAVALGLTLFALQTKYDFTMCGGVLVACLVVFIIFGIIAIFIPGKVIGLVYASLGA 184

DIAVI029891_Dvv FLFCLFIVVDTQMIMK-----TLSPEEYILATINLYMDIINLFVYILRILQELNRQ* 250

XP_028155171.1_Dvv FLFCLFIVVDTQMIMK-----TLSPEEYILATINLYMDIINLFVYILRILQELNRQ- 250

XP_969476.1_Tc FLFCLFIIFDTQMMMQ-----TLSAEEYILATINLYLDIINLFLYILRILQAMNRQ- 250

XP_018567206.1_Ag LVFSLFIIFDTQLIMK-----TLSPEEYILATINLYMDIVNLFLYILRILQAVNRQ- 249

XP_023024494.1_Lc LVFCLFIIFDTQMIMK-----TLSPEEYILATINLYMDIINLFIYILRILQELNRN- 249

XP_019762126.1_Dp FLFCLFIIYDSKLIME-----TLSPEEYILATINLYMDIINLFIYILRILQALNRQ- 288

NP_610824.1_DmA LLFSVYLVYDTQLMLGGNHKYSISPEEYIFAALNLYLDIINIFMYILTIIGLSRN-- 239

NP_725236.1_DmB LLFSVYLVYDTQLMLGGNHKYSISPEEYIFAALNLYLDIINIFMYILTIIGLSRN-- 244

NP_001260927.1_DmC LLFSVYLVYDTQLMLGGNHKYSISPEEYIFAALNLYLDIINIFMYILTIIGLSRN-- 203

NP_001260928.1_DmD LLFSVYLVYDTQLMLGGNHKYSISPEEYIFAALNLYLDIINIFMYILTIIGLSRN-- 239
