# Supplementary material for: Up-regulation of apoptotic- and cell survival-related gene pathways following exposures of western corn rootworm to B. thuringiensis crystalline pesticidal proteins in transgenic maize roots
Source: BMC Genomics. 2021 Sep 4;22:639. doi: 10.1186/s12864-021-07932-4 (PMC8418000; doi:10.1186/s12864-021-07932-4)

**Supplementary Figure S7:** Orthology of stress-induced endoplasmic reticulum protein 2 (SERP2) encoded by the differentially expressed transcript DIAVI057195 in *Diabrotica virgifera virgifera* larvae exposed to Cry3Bb1 and Gpp34/Tpp35Ab1.

**A)** Multiple sequence alignment of SERP2-like proteins with putative orthologs encoded by *D. v. virgifera* gene model XP_028135864.1, and transcript DIAVI057195 that is significantly up-regulated in Cry3Bb1 and Gpp34/Tpp35Ab1 treatments. Accessions provided for representative sequences from arthropods *D. v. virgifera* (Dvv), *Tribolium castaneum* (Tc), *Acyrthosiphon pisum* (Ap), *Anoplophora glabripennis* (Ag), *Blattella germanica* (Bg), *Dendroctonus ponderosae* (Dp), *Drosophila melanogaster* (Dm), *Helicoverpa armigera* (Ha*), Ixodes scapularis* (Is), and *Leptinotarsa decemlineata* (Ld), and mammal *Homo sapiens* (Hs). Residue conservation of 100% (dark grey) and ≥50% (light grey) are indicated in the alignment with ≥64.06% identity.

DIAVI057195 MAPKQRMRLANEKAMKNVTLRGNVPKSTKQ-SQESSPVGPWLLALFVFVVCGSAVFQIIQSIRMA- 64

XP_028135864.1_Dvv MAPKQRMRLANEKAMKNVTLRGNVPKSTKQ-SQESSPVGPWLLALFVFVVCGSAVFQIIQSIRMA- 64

XP_970109.1_Tc MAPKQRMRFANEKASKNVVLRGNVPKSSKQ-QSENSPVGPMILALFIFVVCGSAIFQIIQSIRMA- 64

XP_018569202.1_Ag MAPKQRMRIANEKAMKNVTLRGNVPKSSQKPNQDGPAVGPWLLALFIFVVCGSAVFQIIQSIRLA- 65

XP_019766405.1_Dp MAPKQRMRIANQKASKMVTMRGNVPKSSKQ-EKESSPVGPWLLALFLFVVCGSAVFQIIQSIRMA- 64

XP_023028512.1_Ld MAPKQRMRIANEKAMKNVTLRGNVPKSTKP-SQDGPAVGPWLLALFVFVVCGSAVFQIIQSIRMA- 64

NP_728830.1_Dmel MAPPQRMRVANEKASKYVTMRGNVPKSSKT-KEGQYPVGPWLLALFIFVVCGSAIFQIVQSIRAA- 64

XP_021195434.1_Ha MAPKQRMRIANEIASKNITMRGNVPKSTKE-KDDQYPVAPWLLALFIFVVCGSAVFQIIQSIRLA- 64

AMO26213.1_Bg MAPKQRMRIANEKASKNVTMRGNVPKSSKP-QDEKYPVGPGLLALFIFVVCGSAVFQIIQSIRAA- 64

XP_008184964.1_Ap MAPKQRMRIANEKASKNITQRGNVPKSTKS-EKNKTPVSPWLLALFLFVVCGSVIFQLIQSIRSA- 64

AAY66941.1_Is MVNSQRMRMANEKASKNVVLRGNVPKTTKP-QDEKYPVGPWLLAFFIFVVCGSAIFQIIQSIRFG- 64

NP_001010897.1_Hs MVAKQRIRMANEKHSKNITQRGNVAKTLRP-QEEKYPVGPWLLALFVFVVCGSAIFQIIQSIRMGM 65

*. **:*.**: * :. **** *: : .. *.* :**:*:******.:**::**** .

**B)** Phylogenetic relationship and orthology of putative *Diabrotica virgifera virgifera* SERP2. Maximum-likelihood (ML) analysis of the full SERP2 amino acid sequence of the *D. v. virgifera* DIAVI057195 transcript differentially expressed in Cry3Bb1 and Cry34/35Ab1 treatments and corresponding gene model XP 028135864.1 from the draft genome assembly Dvir_v2 (GenBank Accession PXJM00000000.2) were included. Species abbreviations for sequence accessions are provided in Supplementary Figure 8A. The tree was constructed tree using the LG + G model of protein sequence evolution (Le and Gascuel 2008) which maximized the BIC score (1388.476), and used an empirically-determined gamma shape parameter (*G*) = 0.6295. The consensus tree is shown that minimized the log likelihood score at -625.97 and resulted in a tree with a total branch length of 1.445. Proportional node support obtained using 1,000 bootstrap pseudo-replications is shown for the aligned protein sequence data.


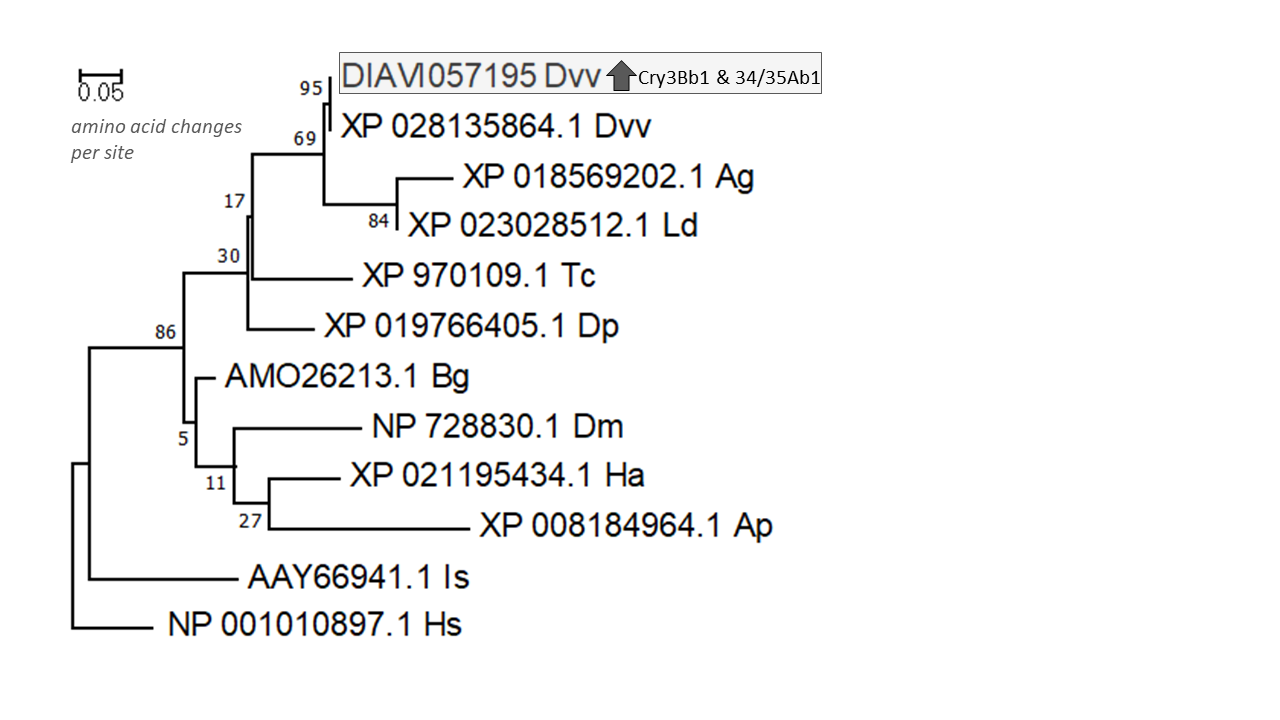

Supplement: Supplementary file 16 — Additional file 16: Supplementary Fig. S7. Orthology of stress-induced endoplasmic reticulum protein 2 (SERP2) encoded by the differentially expressed transcript DIAVI057195 in Diabrotica virgifera virgifera larvae exposed to Cry3Bb1 and Gpp34/Tpp35Ab1. [file 12864_2021_7932_MOESM16_ESM.docx]
